# Transgenic Expression of MicroRNA-181d Augments the Stress-Sensitivity of CD4^+^CD8^+^ Thymocytes

**DOI:** 10.1371/journal.pone.0085274

**Published:** 2014-01-09

**Authors:** Serkan Belkaya, Nicolai S. C. van Oers

**Affiliations:** 1 Department of Immunology, The University of Texas Southwestern Medical Center, Dallas, Texas, United States of America; 2 Department of Pediatrics, The University of Texas Southwestern Medical Center, Dallas, Texas, United States of America; 3 Department of Microbiology, The University of Texas Southwestern Medical Center, Dallas, Texas, United States of America; Maisonneuve-Rosemont Hospital, Canada

## Abstract

Physiological stress resulting from infections, trauma, surgery, alcoholism, malnutrition, and/or pregnancy results in a substantial depletion of immature CD4^+^CD8^+^ thymocytes. We previously identified 18 distinct stress-responsive microRNAs (miRs) in the thymus upon systemic stress induced by lipopolysaccharide (LPS) or the synthetic glucocorticoid, dexamethasone (Dex). MiRs are short, non-coding RNAs that play critical roles in the immune system by targeting diverse mRNAs, suggesting that their modulation in the thymus in response to stress could impact thymopoiesis. MiR-181d is one such stress-responsive miR, exhibiting a 15-fold down-regulation in expression. We utilized both transgenic and gene-targeting approaches to study the impact of miR-181d on thymopoiesis under normal and stress conditions. The over-expression of miR-181d in developing thymocytes reduced the total number of immature CD4^+^CD8^+^ thymocytes. LPS or Dex injections caused a 4-fold greater loss of these cells when compared with the wild type controls. A knockout mouse was developed to selectively eliminate miR-181d, leaving the closely spaced and contiguous family member miR-181c intact. The targeted elimination of just miR-181d resulted in a thymus stress-responsiveness similar to wild-type mice. These experiments suggest that one or more of three other miR-181 family members have overlapping or compensatory functions. Gene expression comparisons of thymocytes from the wild type versus transgenic mice indicated that miR-181d targets a number of stress, metabolic, and signaling pathways. These findings demonstrate that selected miRs enhance stress-mediated thymic involution *in vivo*.

## Introduction

The thymus is the primary organ responsible for T cell development, providing a continuous output of effector and regulatory T cells. Interestingly, this tissue is hyper-responsive to stress, resulting from infections, trauma, pregnancy, starvation, and alcoholism [1], [2], [3], [4], [5], [6], [7]. These diverse forms of stress induce a thymic involution, caused by the deletion of immature CD4^+^CD8^+^ thymocytes and a ensuing reduction in thymic cellularity [8], [9]. In the case of infections, the release of pathogen-associated molecular patterns, such as lipopolysaccharide (LPS), activates Toll-like receptor signaling pathways, releasing inflammatory cytokines that cause thymocyte cell death [10], [11], [12]. Elevations in a subset of these inflammatory cytokines (IL-1β, IL-6, and LIF) induce the production and release of glucocorticoids (GC) via both the hypothalamus-pituitary-adrenal axis and within the thymus itself [13], [14], [15], [16], [17]. The GCs, as lipophilic steroids, diffuse across the plasma membrane and trigger apoptosis of thymocytes by binding to GC-receptors (NR3C1) that are expressed at high levels in the CD4^+^CD8^+^ (DP) thymocytes [18], [19], [20]. Synthetic glucocorticoids (e.g. Prednisone and Dexamethasone) are widely used for the treatment of patients with malignancies and autoimmune diseases, although their effects on thymocytes are not often realized [21], [22]. A second mechanism underlying the stress-induced thymic atrophy is the direct sensing of microbial molecules by pattern-recognition receptors expressed on thymic epithelial cells (TECs) [8], [23], [24]. Activation of these pathways reduces the ability of TECs to support thymopoiesis [8], [25].

Several microRNAs (miRs) can modulate stress responses in tissues such as the thymus, heart, and brain [26], [27], [28]. MiRs are a class of small, non-coding RNA molecules that regulate gene expression at the post-transcriptional level by mRNA degradation and/or translational repression [29], [30]. In the thymus, reductions in the pre-miR RNAse, Dicer, and/or just miR-29a increase the levels of the interferon-alpha receptor (IFNAR) on TECs, decreasing their ability to support thymopoiesis following viral infections [25]. LPS and/or dexamethasone treatments cause a transient loss of both Dicer and Dgcr8 in immature thymocytes within the first 6–12 hours, significantly reducing miR biogenesis [31]. Two-three days after LPS or dexamethasone exposure, there is a selective up- and down-modulation of 7 and 11 thymically-encoded stress responsive miRs, respectively [11]. MiR-181d is one of the most stress-responsive miRs identified in the thymus, declining 15-fold at several days post LPS injection [11]. It is a member of miR-181 family that includes miR-181a, miR-181b, and miR-181c. These four miRs are produced from three different polycistronic clusters: 181ab1, 181ab2, and 181cd [32], [33]. In contrast to the stress effects on miR-181d, miR-181c remains unchanged while miR-181a and miR-181b are reduced 2- and 6-fold, respectively [11]. Such results reveal a differential regulation of miR-181 family members under both steady and disease states [11], [34], [35]. Reductions in miR-181a increase the cell survival of astrocytes from ischemia-like injury following glucose deprivation, in part via elevations in one of its targets, Bcl2 [36]. In developing thymocytes, miR-181a controls T-cell repertoire selection by targeting CD69, Bcl2, Dusp5, Dusp6, Shp2, Ptpn22, and Pten, the protein products of which regulate signaling pathways [31], [36], [37], [38], [39], [40], [41]. While miR-181a/b knock-out (KO) mice have normal αβ T cell development, their NK T cell development is blocked [39], [41]. Contrasting this, the complete deficiency of all miR-181 family members is embryonic lethal, suggesting a functional compensation or redundancy [38].

To study the contribution of miR-181d in stress-induced thymic atrophy, we generated two transgenic (Tg) mouse models with increasing levels of miR-181d expression in immature thymocytes and peripheral T cells. The miR-181d Tg mice exhibited a statistically significant reduction in DP thymocytes. *In vivo* LPS and Dexamethasone (Dex) injections caused a substantial increase in the stress-sensitivity of the DP thymocytes with elevated miR-181d levels. The targeted mutation of the miR-181d sequence in the mouse genome revealed a similar stress-mediated apoptosis as normal mice. These results suggest that multiple miR-181 family members function in a compensatory manner.

## Results

### Generation of miR-181d transgenic mice

**Figure 1:**
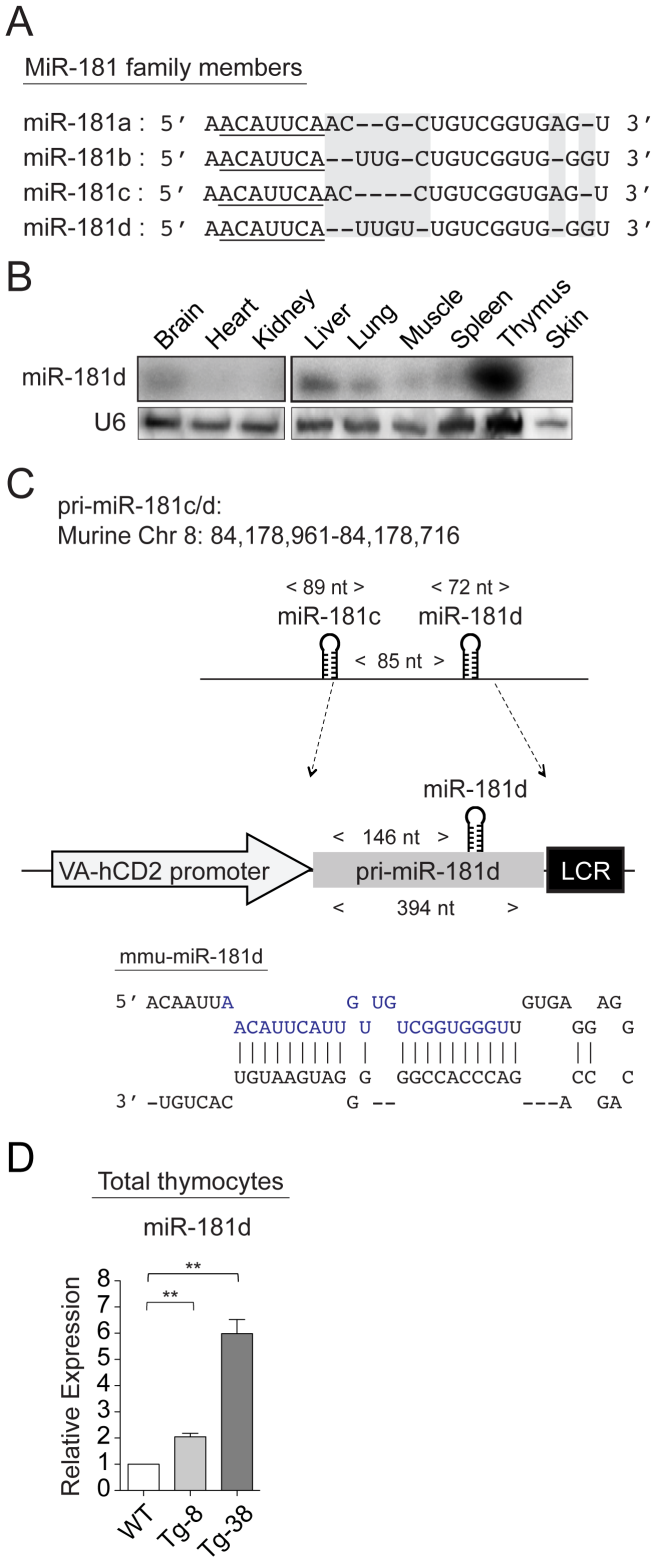
MiR-181d transgenic mice. (A) Schematic shows the sequence homology between mature miR-181 family members. 5′-seed region is underlined. Base differences are shaded with gray. (B) MiR-181d expression in various tissues examined by Northern blotting. U6 probe was used as the endogenous control. (C) Cloning of the pri-miR-181d into the VA-hCD2 transgenic cassette. Stem-loop structure of pre-miR-181d is shown, in which mature miR-181d is highlighted in blue. (D) Relative miR-181d levels were determined by real-time quantitative PCR. Littermate control values were set to 1. Graph represents the mean fold changes +/− SEM normalized to the U6 levels from 3 independent samples, performed in triplicates (n.s.  =  non-significant, *p<0.05, **p<0.01, ***p<0.001; Two-tailed unpaired Student's *t*-test).

The miR-181 family comprises four members, miR-181a, miR-181b, miR-181c, and miR-181d, which are generated from three separate genomic clusters (miR-181ab1, miR-181ab2, and miR-181cd) [32], [33]. While all share an identical seed sequence at their 5′ ends, miR-181d is the most divergent member, differing from the others by 1 to 5 nucleotides (Figure 1A). All miR-181 family members are primarily expressed in the thymus, at levels at least 10-20 fold higher than the brain and liver [35]. In most other tissues, they were very low or undetectable (Figure 1B). Although miR-181c and miR-181d are transcribed from the same cistron, miR-181d is expressed at least 5-10 fold higher in the hematopoietic lineages, including immature thymocytes and T-helper cells [34], [35]. It is one of the most stress responsive miRs in the thymus, with reductions of 15-fold occurring following LPS treatment. MiR-181c expression was unaffected upon stress [11]. This indicates that additional post-transcriptional mechanisms exist for the processing of miR-181d.

In order to determine the contribution of miR-181d to thymopoiesis under normal and stress conditions, we utilized first a gain-of-function approach. Since miR-181c and miR-181d are separated by only 85 nucleotides, the expression of miR-181d could only be achieved by including 146 bases upstream of miR-181d [11]. This excluded the first 28 nucleotides of miR-181c, eliminating the sense-antisense base pairing involved in pre-miR formation, thereby preventing miR-181c over-expression (Figure 1C and Figure S1). With this construct, transgenic mice were generated in which the murine pri-miR181d was expressed in thymocytes and peripheral T cells (Figure 1C) [42]. Two transgenic lines (Tg-8 and Tg-38) were selected based on their increasing levels of miR-181d expression. Relative to the wild-type control, which was set as 1, miR-181d was over-expressed 2- and 6-fold in Tg-8 and Tg-38 lines, respectively (Figure 1D).

### Elevated levels of miR-181d perturb T cell development

**Figure 2:**
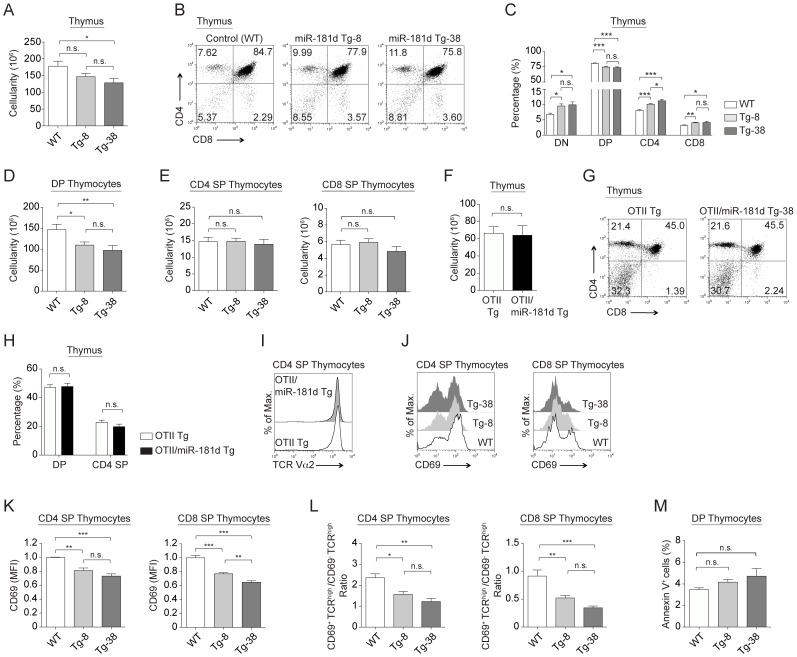
MiR-181d over-expression reduces the number of DP thymocytes. (A) Total thymus cellularity in the control and miR-181d Tg mice. (B) Representative plots show CD4 by CD8 profiles of thymocytes in the control and miR-181d Tg mice, analyzed by FACS. (C) Average percentages of thymocyte subsets (DN, DP, CD4 SP, and CD8 SP) from the control and miR-181d Tg mice. (D) Absolute cell numbers of DP thymocytes. (E) Absolute cell numbers of CD4 SP (left) and CD8 SP (right) thymocytes. (A–E) Data are from WT (n = 18), Tg-8 (n = 25), and Tg-38 (n = 16) mice. (F) Total thymus cellularity of the OTII Tg and OTII/miR-181d Tg-38 mice. (G) Total thymocytes were stained for CD4 and CD8, and analyzed by FACS. (H) Average percentages of DP and CD4 SP thymocytes are shown. (I) Histogram shows the surface expression of TCR (TCR Vα2) gated on CD4^+^CD8^−^ SP thymocytes from the OTII Tg (dark gray) and OTII/miR-181d Tg-38 mice (black line). (F–I) Data are from at least 2 mice per group. Each bar is the mean +/− SEM (n.s.  =  non-significant, *p<0.05, **p<0.01, ***p<0.001; Two-tailed unpaired Student's *t*-test). (J) Histograms show CD69 expression on CD4 SP and CD8 SP thymocytes from the WT (white), Tg-8 (light gray), and Tg-38 (dark gray) mice. (K) Relative MFI (Mean Fluorescence Intensity) levels of CD69 on SP thymocytes. (L) Ratio of the CD69^+^TCRβ^high^ to CD69^−^TCRβ^high^ thymocyte numbers shown for CD4 SP and CD8 SP thymocytes. (M) Average percentages of Annexin V^+^ cells gated on DP thymocytes. (J-M) Data are of at least 3 mice per group. All bar graphs represent the mean +/− SEM values (n.s.  =  non-significant, *p<0.05, **p<0.01, ***p<0.001; One-way ANOVA followed by Tukey's post-hoc test).

The total thymic cellularity was decreased only in the Tg-38 line compared to normal controls, which was similar to the Tg-8 line (Figure 2A). There was an increased percentage of CD4^−^CD8^−^ (DN) cells, with elevated levels of miR-181d (Figure 2B–C). Both the percentage and number of CD4^+^CD8^+^ (DP) thymocytes in Tg-8 and Tg-38 lines were significantly lower than in control mice (Figure 2B–D). While the percentages of CD4^+^CD8^−^ (CD4 SP) and CD4^−^CD8^+^ (CD8 SP) thymocytes were increased significantly, their overall cell numbers were similar, reflecting the decreased percentage of DP thymocytes (Figure 2C, 2E). The DN cells were next characterized for CD44 and CD25 expression, markers used to define 4 subsets, DN1-DN4. The miR-181d transgenic mice had a similar profile of DN1-DN4 cells as wild type mice (Figure S2A). In addition, similar levels of intracellular TCRβ and surface CD5 expression were detected in the DN3 (CD44^−^CD25^+^) thymocytes from the control and miR-181d Tg mice, indicating normal TCR rearrangements and pre-TCR signaling, respectively (Figure S2B). Finally, the proportion and numbers of γδ T cells and NK1.1^+^ cells were similar (data not shown).

The reduced number DP thymocytes in the miR-181d transgenic mice could be caused by accelerated positive selection, diminished cell survival, and/or increased sensitivity to stress. Positive selection appeared intact as miR-181d transgenic DP thymocytes had a normal expression of CD5, CD69, and TCRβ (Figure S2C). This was further supported with similar numbers of OTII-specific TCR transgenic thymocytes developing in miR-181d Tg-38 lines and OTII Tg parental lines (Figure 2F–H). The CD4^+^CD8^−^ thymocytes in the OTII/miR-181d Tg-38 mice had similar expression levels of transgenic TCRα subunit, consistent with normal positive selection (Fig. 2I). However, the expression of CD69 on CD4 and CD8 SP thymocytes was significantly decreased with increased miR-181d levels (Figure 2J-K). Moreover, the ratio of CD69^+^TCRβ^high^ (early stage) to the CD69^−^TCRβ^high^ (late stage) SP thymocytes was lower in miR-181d Tg mice (Figure 2L). This suggests that elevations in miR-181d levels might alter further maturation and/or egress of SP thymocytes. In contrast, Annexin V and 7-AAD staining of immature thymocytes showed no alterations in cell death of DP thymocytes in the Tg-8 and Tg-38 lines (Figure 2M).

### MiR-181d transgenic mice have slightly reduced peripheral T cell numbers

**Figure 3:**
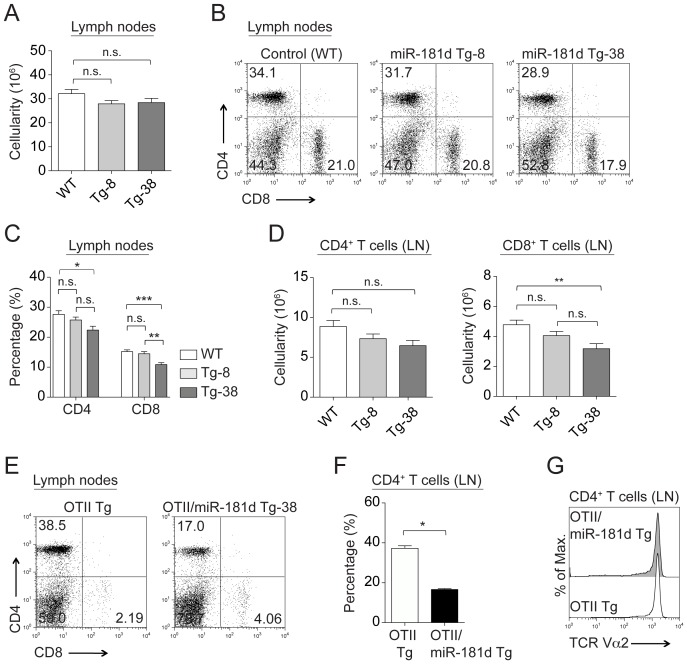
Characterization of peripheral lymphocytes in miR-181d transgenic mice. (A) Total cellularity in the lymph nodes of the control and miR-181d Tg mice. (B) Representative FACS plots of CD4^+^ and CD8^+^ T cells in the lymph nodes. (C–D) Average percentages (C) and absolute numbers (D) of CD4^+^ and CD8^+^ T cells in the lymph nodes. (A–D) Data are of the mean +/− SEM from the WT (n = 17), Tg-8 (n = 23), and Tg-38 (n = 14) mice (n.s.  =  non-significant, *p<0.05, **p<0.01, ***p<0.001; One-way ANOVA followed by Tukey's post-hoc test). (E) CD4 and CD8 profiles of peripheral T cells from the lymph nodes of the OTII Tg and OTII/miR-181d Tg-38 mice. (F) Bar graph shows average percentages of CD4^+^ T lymphocytes in the lymph nodes. (G) Surface expression of TCR (TCR Vα2) gated on CD4^+^ T cells in the lymph nodes of the OTII Tg (dark gray) and OTII/miR-181d Tg-38 mice (black line). (E–G) Data are generated from at least 2 mice per group. Each bar represents the mean +/− SEM values (*p<0.05, **p<0.01, ***p<0.001; Two-tailed unpaired Student's *t*-test).

The total cellularity of lymph nodes and spleen was similar in all the Tg lines compared to normal mice (Figure 3A and Figure S2D). Both percentages and numbers of CD4^+^CD8^−^ T cells were slightly lower with increased miR-181d levels (Figure 3B–D and Figure S2E–G), but this reduction only reached statistical significance in the Tg-38 line when comparing percentages of CD4^+^CD8^−^ T cells in the lymph nodes, and for the absolute numbers of CD4^+^CD8^−^ T cells in the spleen (Figure 2C and Figure S2G). The reductions in peripheral CD4^−^CD8^+^ T cells were more pronounced in miR-181d Tg lines (Figure 3B–D and Figure S2E-G). Both the percentages and numbers of mature CD4^−^CD8^+^ T cells were significantly decreased in lymph nodes and spleen of the Tg-38 line (Figure 3C–D, S2F–G). An even more dramatic reduction in peripheral T cells was noted in the miR-181d Tg-38 lines once crossed onto the OTII TCR Tg line (Figure 3E–F). The TCR density of CD4^+^CD8^−^ T cells remained the same (Figure 3G). While the cellularity was marginally altered, the percentages of peripheral B220^+^ B cells were equivalent in the control and miR-181d Tg mice (Figure S2H). The activation and memory phenotypes were not different when comparing the mice, as revealed with the similar CD44 and CD62L profiles (data not shown). In addition, the naive miR-181d Tg-8 and Tg-38 T cells displayed similar survival and proliferative responses as wild type controls upon anti-CD3/CD28 stimulations *in vitro* (data not shown). Taken together, these results suggested that once the T cells egressed from the thymus, they were functionally normal.

### Transgenic expression of miR-181d augments stress-induced thymic atrophy

**Figure 4:**
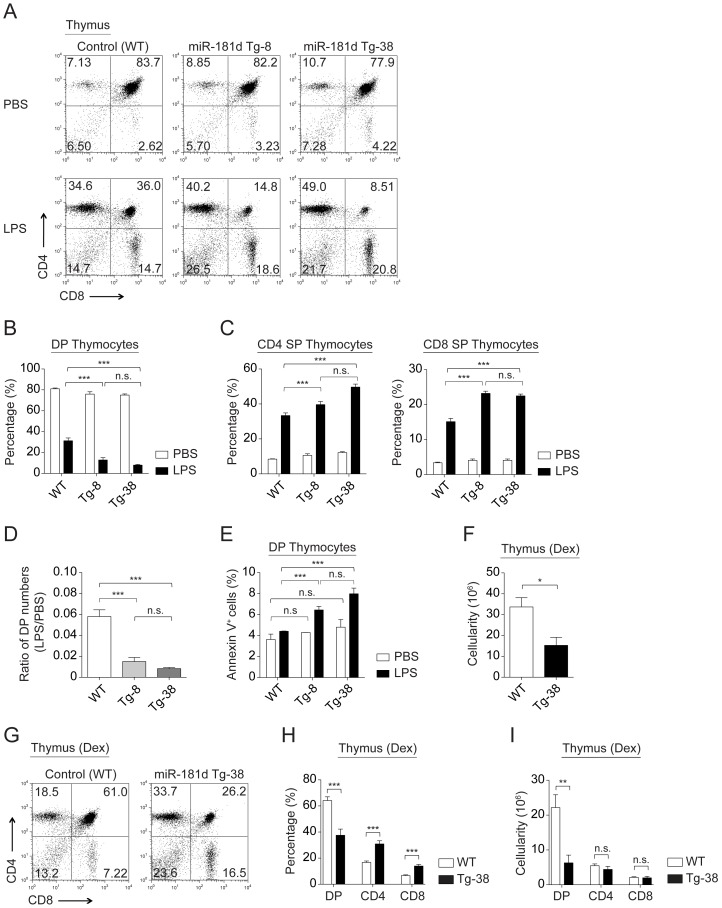
MiR-181d over-expression elevates stress-induced thymic atrophy. (A) Representative plots show CD4 by CD8 profiles of total thymocytes from the control and miR-181d Tg mice at 72 hours after PBS or LPS (100 µg/mouse) injections. (B–C) Graphs demonstrate the average percentages of DP thymocytes (B), and CD4 SP and CD8 SP thymocytes (C) at 72 hours post-injection (PBS, white; LPS, black). (B–C) Data are of the mean +/− SEM from at least 4 independent experiments using at least 3 mice per injection (n.s.  =  non-significant, *p<0.05, **p<0.01, ***p<0.001; Two-way ANOVA followed by Bonferroni's post-hoc test). (D–E) Data were calculated from the experiments shown in the panels A and B. Each bar shows the mean +/− SEM. (D) Ratios of DP thymocyte numbers upon LPS treatment to the numbers of DP thymocytes upon PBS treatment (n.s.  =  non-significant, *p<0.05, **p<0.01, ***p<0.001; One-way ANOVA followed by Tukey's post-hoc test). (E) Average percentages of Annexin V^+^ cells gated on DP thymocytes at 72 hours post-injection (PBS, white; LPS, black). (n.s.  =  non-significant, *p<0.05, **p<0.01, ***p<0.001; Two-way ANOVA followed by Bonferroni's post-hoc test). (F) Total thymic cellularity in the control and miR-181d Tg-38 mice at 48 hours upon Dex injection (60 µg/mouse). (G) Representative FACS plots show CD4 by CD8 profiles of thymocytes after 48 hours post-Dex injection. (H–I) Average percentages (H) and absolute numbers (I) of thymocyte subsets following Dex treatment at 48 hours. (F–I) Bar graphs show the mean +/− SEM from at least 4 mice per treatment (n.s.  =  non-significant, *p<0.05, **p<0.01, ***p<0.001; Two-tailed unpaired Student's *t*-test).

To study the impact of miR-181d on stress-induced thymic atrophy, we analyzed the effects of LPS injections on thymic cellularity. LPS treatment (100 µg/mouse) resulted in 2- and 4-fold greater reduction in both percentages and numbers of DP thymocytes in the Tg-8 and Tg-38 lines, respectively, compared to the wild-type control (Figure 4A–B and Figure S3A–B). A dose response analysis using varying amounts LPS indicated an accelerated depletion of DP thymocytes at all doses (Figure S3C). While the percentages of CD4 SP and CD8 SP thymocytes were increased in the transgenic lines after LPS injection, the absolute numbers of these SP thymocytes remained equivalent to the wild type control (Figure 4C and Figure S3B). The decreased ratio of DP thymocyte numbers in LPS- vs PBS-treated transgenic mice further supported the findings that miR-181d enhanced stress sensitivity of thymocytes (Figure 4D). The DP thymocytes in the miR-181d Tg lines had elevated cell death markers upon stress (Figure 4E). Peripheral T cell numbers were similar in PBS- and LPS-injected miR-181d Tg mice, indicating that the miR-181d effects are specific to the thymus (data not shown) [11].

Consistent with LPS-induced thymic atrophy, an IP injection of dexamethasone (Dex), a synthetic glucocorticoid, also results in a dramatic elimination of the DP thymocytes [11], [31]. Forty-eight hours after Dex injection (60 µg/mouse), Tg-38 mice had more than 2-fold reduction in total thymic cellularity and DP thymocyte numbers (Figure 4F–I). Taken together, these findings indicate that miR-181d over-expression selectively elevates the stress-sensitivity of DP thymocytes.

### T cell development and stress-responses in miR-181d-deficient mice are normal

**Figure 5:**
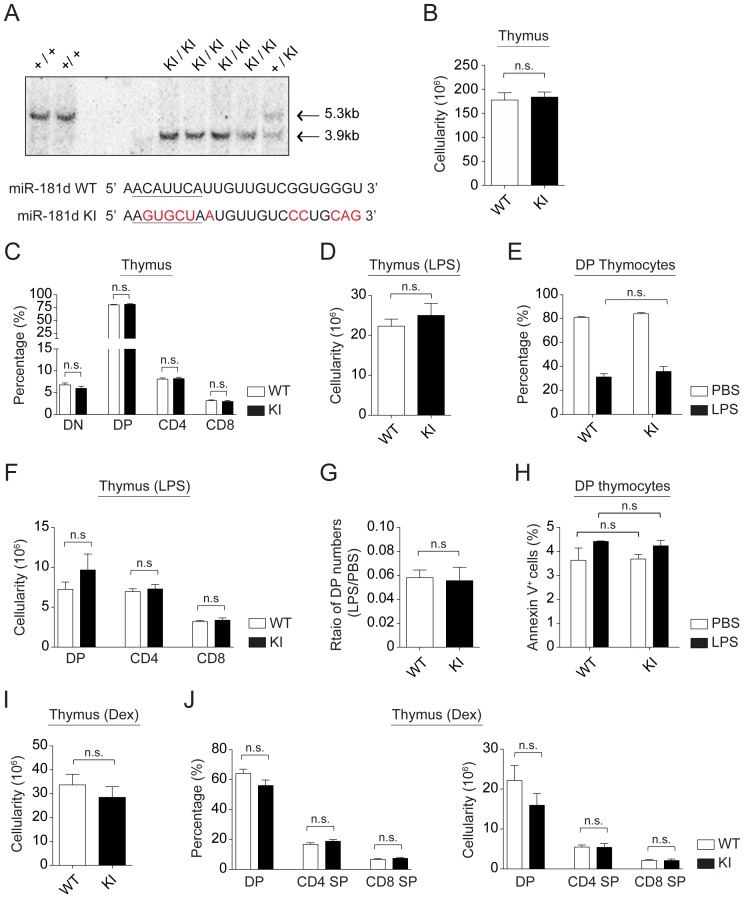
T cell development is normal in miR-181d knock-in mice. (A) Confirmation of miR-181d KI by a representative southern blot. Comparison of the wild type and mutated (miR-181d KI) sequences are provided. 5′-seed region is underlined. Base replacements are highlighted in red. (B) Total thymus cellularity in the control and miR-181d KI mice. (C) Average percentages of thymocyte subsets (DN, DP, CD4 SP, and CD8 SP) are shown for the WT (white) and miR-181d KI (black) mice. (B–C) Data are of the mean +/− SEM from the WT (n = 18) and miR-181d KI (n = 17) mice. (D) Total thymus cellularity in the control and miR-181d KI mice at 72 hours post-LPS (100 µg/mouse) injection (n.s.  =  non-significant; Two-tailed unpaired Student's *t*-test). (E) Average percentages of DP thymocytes at 72 hours after PBS or LPS treatment (n.s.  =  non-significant; Two-way ANOVA followed by Bonferroni's post-hoc test). (F) Absolute cell numbers of thymocyte subsets at 72 hours post-LPS injection (n.s.  =  non-significant; Two-tailed unpaired Student's *t*-test). (D–F) Data show the mean +/− SEM at least 4 independent experiments using at least 3 mice per treatment. (G–H) Data were calculated from the experiments shown in the panels D and E. Each bar shows the mean +/− SEM. (G) Ratios of DP thymocyte numbers upon LPS treatment to the numbers of DP thymocytes upon PBS treatment (n.s.  =  non-significant; Two-tailed unpaired Student's *t*-test). (H) Average percentages of Annexin V^+^ cells gated on DP thymocytes at 72 hours post-injection (PBS, white; LPS, black). (n.s.  =  non-significant; Two-way ANOVA followed by Bonferroni's post-hoc test). (I) Total thymic cellularity in the control and miR-181d KI mice at 48 hours upon Dex injection (60 µg/mouse). (J) Average percentages (left) and absolute numbers (right) of thymocyte subsets following Dex treatment at 48 hours. (I–J) Bar graphs show the mean +/− SEM from at least 4 mice per treatment (n.s.  =  non-significant; Two-tailed unpaired Student's *t*-test).

To further define the functional role of miR-181d in the stress response, we generated a miR-181d knock-out line. Since miR-181c and miR-181d are separated by only 85 nucleotides, we utilized a knock-in (KI) approach in which only the miR-181d sequence was modified (miR-181d KI) (Figure 5A and Figure S4). A total of 11 base-replacements (five in the 5′ seed region) were introduced into the miR-181d sequence. This was done to disrupt the formation and processing of the pre-miR-181d stem-loop structure, without affecting miR-181c (Figure 5A and Figure S5). Sequencing reactions confirmed the KI status of the locus (data not shown). Initial Northern blotting experiments revealed a very faint signal for miR-181d in miR-181d KI thymocytes (data not shown). This was a consequence of the miR-181d probe binding to the endogenous mature miR-181b, which differs by only 1 nucleotide compared to miR-181d. To further confirm that miR-181d was not expressed with the KI design, plasmid constructs containing the mutated miR-181d (KI) sequences were transfected into HEK293T cells. None of the miR-181 family members are normally expressed in these cells [11]. Subsequent Northern blotting showed that the mutations in miR-181d prevented the expression of mature miR-181d, which could only be detected with the wild type miR-181d expression vector (Figure S6A). Of note, miR-181d*, the passenger strand of miR-181d, was not detected in miR-181d KI thymocytes (data not shown). The miR-181d KI mice had normal T cell development, with similar percentages and numbers of thymocyte subsets when compared with wild type controls (Figure 5B–C and Figure S6B–F). Consistent with the normal thymopoiesis, the number and percentage of peripheral lymphocytes in these mice were also similar to wild-type controls (Figure S6G–I). Of all the cell populations analyzed, the peripheral T cell percentages were significantly elevated in the spleen of miR-181d KI mice compared to wild-type controls (Figure S6H). In addition, naive peripheral miR-181d KI T cells exhibited similar survival and proliferative responses as wild-type controls upon anti-CD3/CD28 stimulations *in vitro* (data not shown). While the transgenic expression of miR-181d augmented stress-induced thymic atrophy, its selective elimination had no effect on DP cell depletion following LPS or Dex injections (Figure 5D–G and Figure 5I–J). Moreover, there was a similar level of Annexin V induction in the KI compared to normal mice in response to stress (Figure 5H). Finally, the percentage and number of SP thymocytes appeared normal in the miR-181d KI mice following LPS and Dex treatments (Figure 5F and Figure 5J). These experiments suggest that the targeted elimination of one miR-181 family member is insufficient to modulate the stress responsiveness of developing thymocytes.

### Analysis of differential gene expression in miR-181d transgenic thymocytes

**Figure 6:**
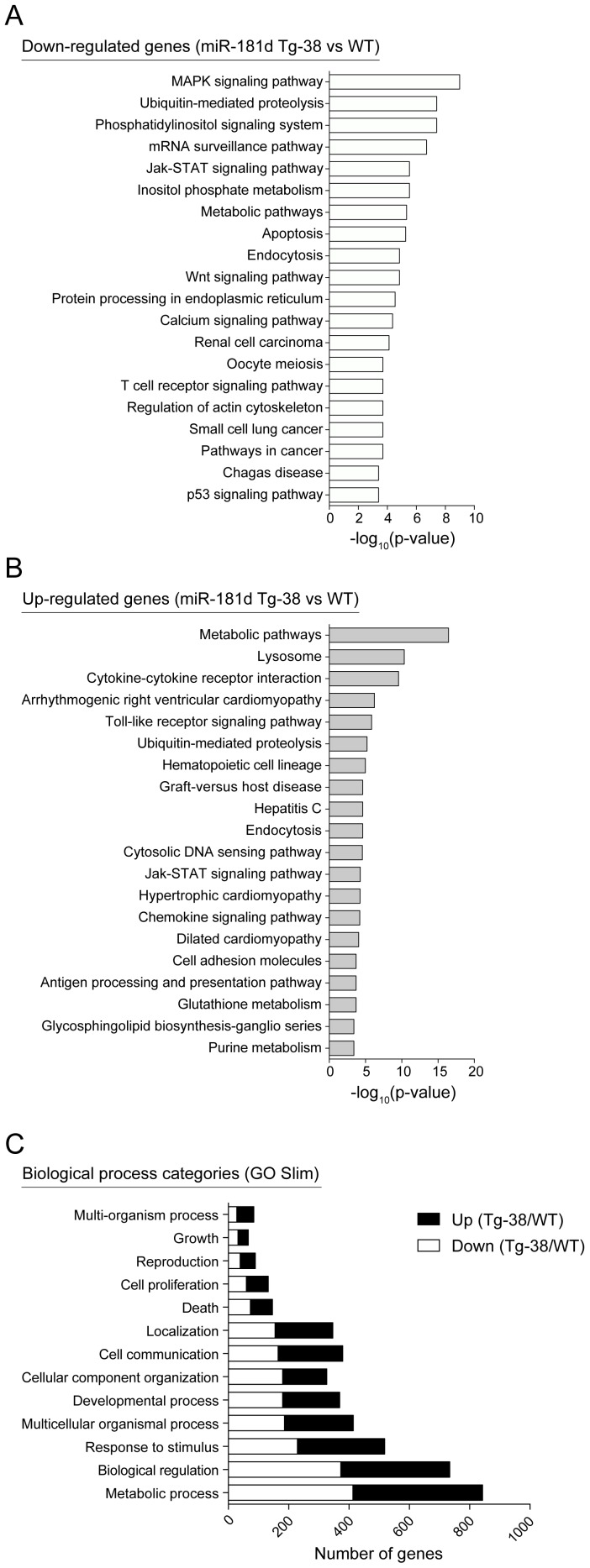
KEGG pathway and Gene ontology analyses of differentially regulated genes in miR-181d transgenic thymocytes. (A–B) Top 20 over-represented KEGG pathways are shown based on the statistical significance for down-regulated (A) and up-regulated genes (B) with more than 1.2-fold (*p*<0.05) in the miR-181d Tg-38 thymocytes compared to the wild type control. Pathway enrichment analysis was performed using the Web-based Gene Set Analysis Toolkit. (C) Biological process categories over-represented within the dysregulated genes are shown. White and black bars are of down- and up-regulated genes in the miR-181d Tg-38 thymocytes, respectively. Gene ontology Slim (GO Slim) analysis was performed using the Web-based Gene Set Analysis Toolkit.

While a number of mRNA targets of miR-181 have been reported, it is not known whether miR-181d has overlapping and/or distinct targets. Therefore, gene expression comparisons were done with the wild-type control and miR-181d Tg-38 mice. Of the 26,000 genes probed on the array, 111 were down- and 237 were up- regulated more than 1.5-fold in the thymus of miR-181d Tg-38 mice compared to the wild type control (*p*<0.05) (Table S1 and Table S2). KEGG Pathway Analysis was applied to the genes significantly modulated more than 1.2-fold. The top 20 over-represented canonical pathways are listed for both down- and up-regulated genes (Figure 6A–B). The most significant pathways affiliated with down-regulated genes included MAPK signaling, phosphatidylinositol signaling, calcium signaling, TCR signaling, and apoptotic pathways. Jak-STAT signaling, ubiquitin-mediated proteolysis, and metabolic pathways were significantly enriched both among the down- and up-regulated genes in miR-181d Tg thymus (Figure 6A–B). We also performed Gene Ontology Slim (GO Slim) analysis with the Web-based Gene Set Analysis Toolkit (WebGestalt) to obtain a broad summary of the dysregulated genes (miR-181d Tg vs wild type thymocytes) [43], [44]. GO Slim classification was provided with the number of genes for each biological process category (Figure 6C). Most of the up- and down-regulated genes in miR-181d Tg thymocytes were represented within the metabolic process category (Figure 6C). These data indicate the involvement of miR-181d-targeted genes in cell metabolism and stress responses, consistent with the phenotypes revealed in the Tg mice.

We next performed Transcription Factor (TF) Target enrichment through the WebGestalt, to identify the genes sharing similar TF target motifs among the dysregulated genes in the wild type vs miR-181d Tg thymocytes (Table S3). A significant number of these genes had predicted binding sites for Foxo4 and Myc, both of which are direct targets of the PI3K/Akt signaling pathways [45], [46], [47].

## Discussion

MiR-181d is one of the most down-regulated miRs detected in the thymus following stress [11]. We used both transgenic and gene targeting approaches in mouse models to determine the role of miR-181d in thymopoiesis under normal and stress conditions. While the transgenic over-expression of miR-181d resulted in a slight reduction in CD4^+^CD8^+^ (DP) thymocytes, without an impairment of TCR-driven positive selection, the depletion of DP thymocytes following LPS or Dex injections was significantly increased. Such experiments indicate that miR-181d potentiates programmed cell death. This would suggest that the down-modulation of miR-181d occurring following stress could protect DP thymocytes from apoptosis and/or enhance their recovery.

Most DP thymocytes undergo a process of death by neglect, partly through the systemic and intrathymic production of glucocorticoids. Stress elevates these glucocorticoid levels, enhancing the magnitude and kinetics of cell death. Within the first 6-12 hours, stress causes a global reduction in miRs by the degradation of Dicer and Dgcr8 [31]. By 48–72 hours, and once Dicer levels are restored, there is a differential regulation of miRs, some up- and others down-regulated. Interestingly, while miR-181d was down-modulated around 15-fold, the much more abundantly expressed miR-181a and miR-181b family members were only minimally affected [11]. This indicates that the processing of the miR-181c/d locus during stress is very distinct from the two miR-181a/b loci. In fact, the processing appears specific to miR-181d, as miR-181c is only marginally affected in spite of being expressed from the same cistron and separated by only 85 nucleotides.

Most studies to date have focused on miR-181a, the most abundant miR in DP thymocytes [35]. MiR-181a targets mRNAs encoding TCR signaling proteins, thereby controlling repertoire selection by modulating signaling thresholds [40], [48]. Interestingly, a gene expression analysis of mice lacking miR-181a/b revealed a distinct set of targets. These included Pten, a regulator of PI3K/Akt signaling [39]. In our study, the phosphatidylinositol signaling system and metabolic pathways were the most significant pathways enriched among the miR-181d down-regulated genes, consistent with the findings using miR-181a/b-deficient mice. Furthermore, many of the targeted genes had Foxo4 or Myc binding motifs, and these two transcription factors are regulated by PI3K/Akt. Such results strongly suggest that miR-181d targets genes responsible for cell metabolism and survival. Since stress and metabolic rates are intricately linked, the altered expression of miR-181d would modulate energy and nutrient demands within the cell. It is also plausible that stress can lead to a metabolic reprogramming in immature thymocytes by modulating miR-181 levels. This could explain massive loss of DP thymocytes during thymic atrophy via shutting down high-energy consumption processes, such as T cell repertoire selection.

MiR-181a regulates signaling down-stream of Notch1 [37], [38]. Notch1 is a critical regulator of T cell development [49]. In fact, Notch1 signaling increases the resistance of DP thymocytes to GC-induced cell death [50], [51], [52]. Elevations of miR-181d would be expected to attenuate Notch1 signaling, increasing the magnitude of DP cell death in response to stress. MiR-181 family members also target Bcl2, with its reduction increasing the GC-sensitivity of DP thymocytes [19], [36], [53]. Therefore, it is likely that the diverse miR-181d targets in TCR-, PI3K/Akt-, Notch1- and anti-apoptotic pathways combinatorially modulate the stress responses of thymocytes. Indeed, miR functions are not only dependent on cellular concentrations of miRs, but also dependent on the abundance of target mRNAs that can be substantially altered by stress conditions [26], [27]. Thus, miR-181d can have novel and/or additional gene targets in thymocytes upon stress, apart from their validated targets under steady states. This could also account for the increased stress sensitivity of DP thymocytes in miR-181d Tg lines. CD69 is previously reported as one of the overlapping targes of miR-181a and miR-181d [11], [53]. Transgenic expression of miR-181d did not alter the ratio of pre-selection (CD69^−^) and post-selection (CD69^+^) DP thymocytes, but appeared to diminish T cells leaving the thymus by reducing the levels of CD69 expression on SP thymocytes. It is also possible that miR-181d does not target CD69 on DP thymocytes. The additional miR-181d-mediated effects on the SP thymocytes could result from it targeting distinct mRNA species.

To specifically define the role of miR-181d in thymopoiesis, we developed a miR-181d gene-targeted mouse in which the miR-181d seed sequence and hairpin loop were changed. There was no effect of this knockout on either normal or stress-modulated thymopoiesis. This finding is consistent with recent reports that miR-181c/d knock-out mice have normal T cell development [38], [39]. This strongly argues for a functional redundancy/compensatory process among the miR-181 family members. Consistent with this, a complete targeting of all miR-181 family members causes an embryonic lethality [39]. Accordingly, T cell-specific elimination of miR-181 family members might be beneficial to recover from thymic atrophy. In addition to miR-181d, we identified 17 other stress-responsive miRs in the thymus. All have known targets that could influence stress responses, including the miR-17-92a family that targets pro-apoptotic genes [54], [55]. MiR-185 is another stress-responsive thymic miR that is haploinsufficient in 22q11.2 Deletion Syndrome patients and down-regulated following LPS or Dex exposure [11], [56]. Unlike miR-181d, the transgenic over-expression of miR-185 blocks thymopoiesis, leading to a peripheral T cell lymphohenia. Its effects on thymopoiesis are partly via the targeting of of Mzb1 (Marginal zone B and B1 cell-specific protein), NFATc3 (Nuclear factor of activated T-cells, cytoplasmic, calcineurin-dependent 3), and Camk4 (Calcium/Calmodulin-dependent protein kinase type IV) [57]. Such stress-induced down-regulation of miR-185 might be necessary for the survival of DP thymocytes, since its over-expression attenuates proper selection and further differentiation of these cells.

Together with previous reports, our study further supports the involvement of miRs in stress-induced thymic involution. In particular, elevated levels of miR-181d lead to increased loss of DP thymocytes upon stress. This may be advantageous by preventing toleragenic signalings in immature thymocytes to foreign antigens that are introduced with infectious agents. Overall, these findings suggest that miR-181d might be good therapeutic target for hematological malignancies exhibiting resistance to GC-induced apoptosis.

## Materials and Methods

### Ethics Statement

Mouse procedures were carried out in accordance with the Institutional Animal Care and Use Committee (IACUC) at the University of Texas Southwestern Medical Center. The IACUC committee specifically approved this study (IACUC #2010-0053). All animal use adheres to applicable requirements such as the Animal Welfare Act, the Guide for the Care and Use of Laboratory Animals, and the US Government Principals regarding the care and use of animals. The mice were housed in the specific pathogen free facility on the North campus of UT Southwestern Medical Center.

### Mice

The miR-181d transgenic lines were generated by the UT Southwestern Medical Center Transgenic Core facility. The VA-hCD2 transgenic cassette containing a pri-miR-181d genetic fragment of 394 bp was injected into fertilized eggs derived from C57BL/6 mice. This fragment was cloned from genomic DNA, isolated from C57BL/6 mice, using standard PCR reactions [11]. The transgenic construct was designed with the first 28 nucleotides of miR-181c lacking. This eliminates a significant segment of miR-181c, while leaving intact miR-181d. Transgenic founders were identified using DNA probes for the VA-hCD2 transgenic cassette using previously described assays [58]. The expression of miR-181d was subsequently confirmed by RT-PCR techniques and Northern blotting. The OTII transgenic line refers to the T cell receptor transgenic mice with specificity for a peptide derived from ovalbumin presented on major histocompatibility complex class II (MHC class) I-A^b^. OTII/miR-181d double transgenic mice were generated from crosses between the OTII Tg and miR-181d Tg-38 lines.

For the generation of the miR-181d knock-in construct, PCR reactions were performed to amplify a 3.56 kb genomic DNA fragment containing miR-181d followed by miR-181c (reverse orientation). Bam HI and Bgl II restriction sites were incorporated at the 5′ and 3′ ends, respectively. All genomic PCR reactions were undertaken with LA-Taq polymerase (Takara Inc., Thermo-Fisher Scientific), and the constructs were directly cloned into pCR2.1-TOPO-TA cloning vectors according to the manufacturers' instructions (Invitrogen). dsDNA sequencing reactions confirmed nucleotide sequence information. A 3.03 kb genomic piece that continued from miR-181c, included new Nhe I and Hind III restriction sites, was PCR amplified and also cloned into a pCR2.1 TOPO-TA cloning vector. This piece was subcloned into the targeting vector, pGKneoloxP2dta, that was linearized with Hind III (vector was a kind gift from Dr. Toru Miyazaki, University of Tokyo, Japan). Site-directed mutagenesis was used to modify miR-181d, with 11 nucleotide replacements to eliminate the seed region and the hairpin loop. A new Pst I restriction site was cloned into this region. The original miR-181d sequence was acaattaacattcattgttgtcggtgggttgtg and the new mutated sequence was acaattaagtgctaatgttgtcc**ctgcag**tgtg, with the underlined nucleotides changed and the bold region high-lighting the Pst I site. This region was subcloned into the left arm of pGK-neomycin using a Bgl II linearized vector. The pGK-neo-miR-181d knock-in construct was linearized with Not I, purified, and electroporated into C57BL/6-derived embryonic stem cells (LR2.6.1) by the UT Southwestern Medical Center Transgenic and Knock-out Core facility. ES cell clones were selected with G418 and gancyclovir, and correct insertion of the targeted allele was determined by Southern blotting following digestion of ES cell DNA with Xba I. Of the 600 clones screened, 4 ES cell lines that contained the correctly sized targeted allele were identified (6B12, 5C1, 2B7, and 2C9). The wild-type allele is 5.3 kb, while the targeted allele is 3.9 kb. Two of the ES cell lines, 5C1 and 2B7, were separately used for injections into C57BL/6 blastocyts. The resulting chimeric male mice were mated with C57BL/6 female mice. Following subsequent interbreeding between heterozygous mice, homozygous mice (miR-181d KI^neo^) were crossed with CAG-Cre transgenic lines (on a C57BL/6 background), eliminating the neomycin cassette and leaving a loxP site. MiR-181d KI progeny mice were further confirmed for the mutated miR-181d sequence by PCR reactions and subsequent DNA sequencing as well as Southern blotting. The primers used to identify the KI allele were miR181dKI4691 (5′-ccaacacctaaccctccag-3′) and PCRmir181dKI3′ (5′-gtgctaatgttgtccctgc-3′). The miR181d KI line is currently being deposited with the mouse mutant resource center ([C57BL/6-Mir181d tm1Oers/Mmucd, #036959-UCD]).

In order to compare folding structures of wild type miR-181d and miR-181d KI sequences, RNAfold Web Server (http://rna.tbi.univie.ac.at/cgi-bin/RNAfold.cgi) was used to predict Minimum Free Energy (MFI) structures based on the parameters as described previously [59].

Lipopolysaccharide (LPS from E. coli 0111:B4, Sigma L4391) and dexamethasone (Dex, Sigma D2915) were prepared at 1 mg/ml in PBS and at 0.06 mg/ml in water, respectively. Mice 5-8 weeks of age were used in all experiments including intraperitoneal injections of PBS, LPS, and Dex.

### Cell isolation, culture, and flow cytometry

Single cell suspensions were freshly prepared from isolated lymphoid organs, followed by FACS staining as described previously [60]. Total cellularity was determined by counting live cells upon Trypan blue staining. Absolute cell numbers were calculated using total cellularity and percentages of subsets in the lymphoid organs. Unless otherwise indicated, all antibodies for immunostaining used in this study were purchased from BD Biosciences. DN thymocyte subsets were analyzed for CD25 and CD44 expression gated on CD4^−^ CD8^−^ TCRγδ^−^ NK1.1^−^ B220^−^ CD11b^−^ CD11c^−^ thymocytes. Intracellular TCRβstaining was undertaken using Cytoperm/Cytofix Kit (BD Biosciences). Quantification of apoptosis/cell death was assessed by staining with antibodies against Annexin V and 7AAD. Ten thousand to 1×10^6^ cells per sample were acquired on FACSCalibur and LSRII flow cytometers (Becton Dickinson). Data were analyzed using FlowJo software (Tree Star).

Wild-type pri-miR-181d sequence (∼394 bp, excluding the first 28 nucleotides of miR-181c) was cloned into pCDNA3.1 (Invitrogen). The mutated miR-181d (KI) sequence (∼394 bp, excluding the first 28 nucleotides of miR-181c) was amplified by PCR using genomic DNA isolated from a tail biopsy from the miR-181d KI mice. The PCR product was cloned into the pCR2.1-TOPO-TA cloning vector (Invitrogen), followed by subcloning into the pCDNA3.1 vector. Primers used to amplify the wild-type and mutated pri-miR-181d regions were provided as in Figure S1. Transfections were done in HEK293T cells (6-well plate) using the X-tremeGENE 9 DNA Transfection Kit (Roche Applied Science). Totat RNA isolation and subsequent Nothern blotting were performed at 48 hours post-transfection.

### RNA analysis

Total RNA (including microRNAs) was isolated with the miRNeasy kit (Qiagen). For northern blotting, 5-15 µg of total RNA was resolved on 15% urea/polyacrylamide gels and transferred to Zeta probe membranes. Following carbodiimide-mediated cross-linking [61], the membranes were hybridized with miR-181c and miR-181d probes labeled with [^32^P]-dATP using the Starfire kit (Integrated DNA Technologies, Coralville, IA). A U6 probe was used as the endogenous control. Bands were visualized with a phosphorimager (GE Healthcare). For the microRNA real-time PCR, total RNA was treated with DNase (Turbo-DNAse, Ambion). cDNA was made from 10 ng of total RNA using the TaqMan MicroRNA Reverse Transcription Kit (Applied Biosystems). Real-time PCR analysis was performed using TaqMan Gene Expression Master Mix, and miR-181d specific TaqMan probes on an ABI 7300 series PCR machine (Applied Biosystems) according to the manufacturers' recommendations. U6 probe was used as the endogenous control. All real-time quantitative PCR reactions were performed in triplicate. Relative expression of miRs was calculated by the comparative threshold method (ΔΔCT).

### MicroArray analysis

Whole thymus tissues from the wild type control (n = 3) and miR-181d Tg-38 (n = 3) mice were isolated followed by homogenization in Qiazol. RNA was isolated with the Qiagen miRNeasy kit. RNA quality and integrity was examined using Bioanalyzer Chip. cDNA synthesis and hybridization onto Illumina SingleColor MouseWG-6_V2_0_R0_11278593 platform were performed at the UTSW Genomics and Microarray Core Facility. Subsequent analysis of microarray raw data was performed as described previously, followed by associative *t*-test analysis to identify significantly (*p*<0.05) deregulated genes among the wild type and miR-181d Tg samples [56], [62]. Microarray data were submitted to GEO database under accession number GSE51778. KEGG pathway analysis, Gene Ontology Slim classification, and Transcription Factor Target analysis (based on the MsigDB) were performed through the WebGestalt (Web-based Gene Set Analysis Toolokit, http://bioinfor.vanderbilt.edu/webgestalt/). 711 down- and 879 up-regulated genes more than 1.2-fold (*p*<0.05) were used in the enrichment analyses with at least 6 genes for each category through the hyper-geometric test and Benjamini & Hochberg as multiple test adjustment.

### Statistical analyses

Mean values, standard error of the mean (SEM), and statistical analyses were calculated with GraphPad Prism Software. The statistical significance was designated with asterisks (*p<0.05, **p<0.01, ***p<0.001) and *p*-values more than 0.05 were considered non-significant (n.s.).

## Supporting Information

Figure S1 **Generation of the VA-hCD2-pri-miR-181d transgenic cassette.** Pri-miR-181c/d cluster is located on mouse chromosome 8. Lengths of miR-181c (blue) and miR-181d (red) sequences are 89 and 72 nucleotides, respectively. A 394 nt region containing whole pri-miR-181d and a 61nt portion of pri-miR-181c (lacking the seed sequence) was PCR amplified using the primers indicated with arrows and cloned into the VA-hCD2 transgenic cassette through EcoRI sites. Mature miR-181c (blue) and miR-181d (red) sequences are shown in uppercase, indicating that mature miR-181c sequence was excluded from the transgenic cassette.
(TIF)

Figure S2 **Characterization of lymphocytes in miR-181d transgenic mice.** (A) CD25 and CD44 markers were used to define DN subsets by gating on CD4^−^ CD8^−^ B220^−^ NK1.1^−^ TCRγδ^−^ CD11b^−^ and CD11c^−^ thymocytes. Absolute numbers of DN subsets are shown as the mean +/− SEM using at least 6 mice per group. (B) Histograms show intracellular TCRβ and surface CD5 expression in DN3 thymocytes from the WT (white), Tg-8 (blue), and Tg-38 (red) mice. Average percentages of intracellular TCRβ^+^ DN3 thymocytes are provided. (C) Histograms show CD5, CD69, and TCRβ expression on DP thymocytes. (D) Total cellularity in the spleen of the control and miR-181d Tg mice. (E) Representative FACS plots show CD4 by CD8 profiles in the spleen. (F–G) Average percentages (F) and absolute numbers (G) of CD4^+^ and CD8^+^ T cells in the spleen. (D–G) Data are from the WT (n = 16), Tg-8 (n = 16), and Tg-38 (n = 11) mice. (H) Average percentages of B220^+^ B cells in the lymph nodes (left) and spleen (right) using at least 10 mice per group. All bar graphs show the mean +/− SEM (n.s.  =  non-significant, *p<0.05, **p<0.01, ***p<0.001; One-way ANOVA followed by Tukey's post-hoc test).
(TIF)

Figure S3 **Stress-induced thymic atrophy in miR-181d transgenic mice.** (A) Total thymic cellularity in the control and miR-181d Tg mice at 72 hours upon LPS injection (100 µg/mouse). (B) Absolute cellularity of thymocyte subsets (DP, CD4 SP, and CD8 SP) after 72 hours post-LPS injection. (A–B) Data are of the mean +/− SEM from at least 4 independent experiments using at least 3 mice per treatment (n.s.  =  non-significant, *p<0.05, **p<0.01, ***p<0.001; One-way ANOVA followed by Tukey's post-hoc test). (C) FACS plots show CD4 by CD8 profiles in the thymus of the control and miR-181d Tg mice at 72 hours upon LPS injection at varying concentrations (10, 30, and 100 µg/mouse). Percents are provided in each quadrant.
(TIF)

Figure S4 **MiR-181d knock-in strategy.** Schematic represents the generation strategy of miR-181d KI mice.
(TIF)

Figure S5 **Predicted secondary structures of the wild-type miR-181d and miR-181d knock-in sequences.** RNAfold Web Server (http://rna.tbi.univie.ac.at/cgi-bin/RNAfold.cgi) was used to obtain Minimum Free Energy (MFE) structures. Mature mir-181c and miR-181d sequences are highlighted in green and blue, respectively. Mutated bases in the miR-181d knockin sequence are highlighted in red.
(TIF)

Figure S6 **Characterization of miR-181d knock-in mice.** (A) Northern blot shows miR-181d and miR-181c expression in HEK293T cells transfected with pCDNA3.1 control, pCDNA3.1/miR-181d, or pCDNA3.1/miR-181d KI plasmids. A U6 probe was used as endogenous control. Data are representative of 2 independent experiments. (B) Absolute numbers of DN thymocyte subsets in the thymus of the control and miR-181d KI mice. Data are of the mean +/− SEM using at least 6 mice per group (n.s.  =  non-significant; Two-tailed unpaired Student's *t*-test). (C) Histograms show intracellular TCRβ (icTCRβ) and surface CD5 expression in DN3 thymocytes from the WT (white) and miR-181d KI (green) mice. Average percentages of icTCRβ^+^ DN3 thymocytes were provided. (D) Histograms show CD5, CD69, and TCRβ expression gated on DP thymocytes from the WT (white) and miR-181d KI (green) mice. (E) Relative MFI (Mean Fluorescence Intensity) levels of CD69 on SP thymocytes. (F) Ratio of the CD69^+^TCRβ^high^ to CD69^−^TCRβ^high^ thymocyte numbers gated on CD4 SP and CD8 SP thymocytes. (E–F) Data show the mean +/− SEM values from at least 3 mice per group (n.s.  =  non-significant; Two-tailed unpaired Student's *t*-test). (G–H) Average percentages and absolute cell numbers of CD4^+^ T and CD8^+^ T cells in the lymph nodes (G) and spleen (H) of the WT (n = >16) and miR-181d KI (n = >13) mice. (I) Average percentages of B220^+^ B cells in the lymph nodes and spleen using at least 13 mice per group. All bar graphs show the mean +/− SEM (n.s.  =  non-significant; Two-tailed unpaired Student's *t*-test).
(TIF)

Table S1 **List of down-regulated genes more than 1.5-fold in miR-181d Tg-38 thymus compared to the wild type control.**
(PDF)

Table S2 **List of up-regulated genes more than 1.5-fold in miR-181d Tg-38 thymus compared to the wild type control.**
(PDF)

Table S3 **Top 10 transcription factors with predicted target motifs among differentially regulated genes in the wild type control versus miR-181d Tg-38 thymocytes based on the significance level.**
(PDF)
