# Supplementary figures and images for: Transgenic Expression of MicroRNA-181d Augments the Stress-Sensitivity of CD4+CD8+ Thymocytes
Source: PLoS One. 2014 Jan 9;9(1):e85274. doi: 10.1371/journal.pone.0085274 (PMC3887031; doi:10.1371/journal.pone.0085274)

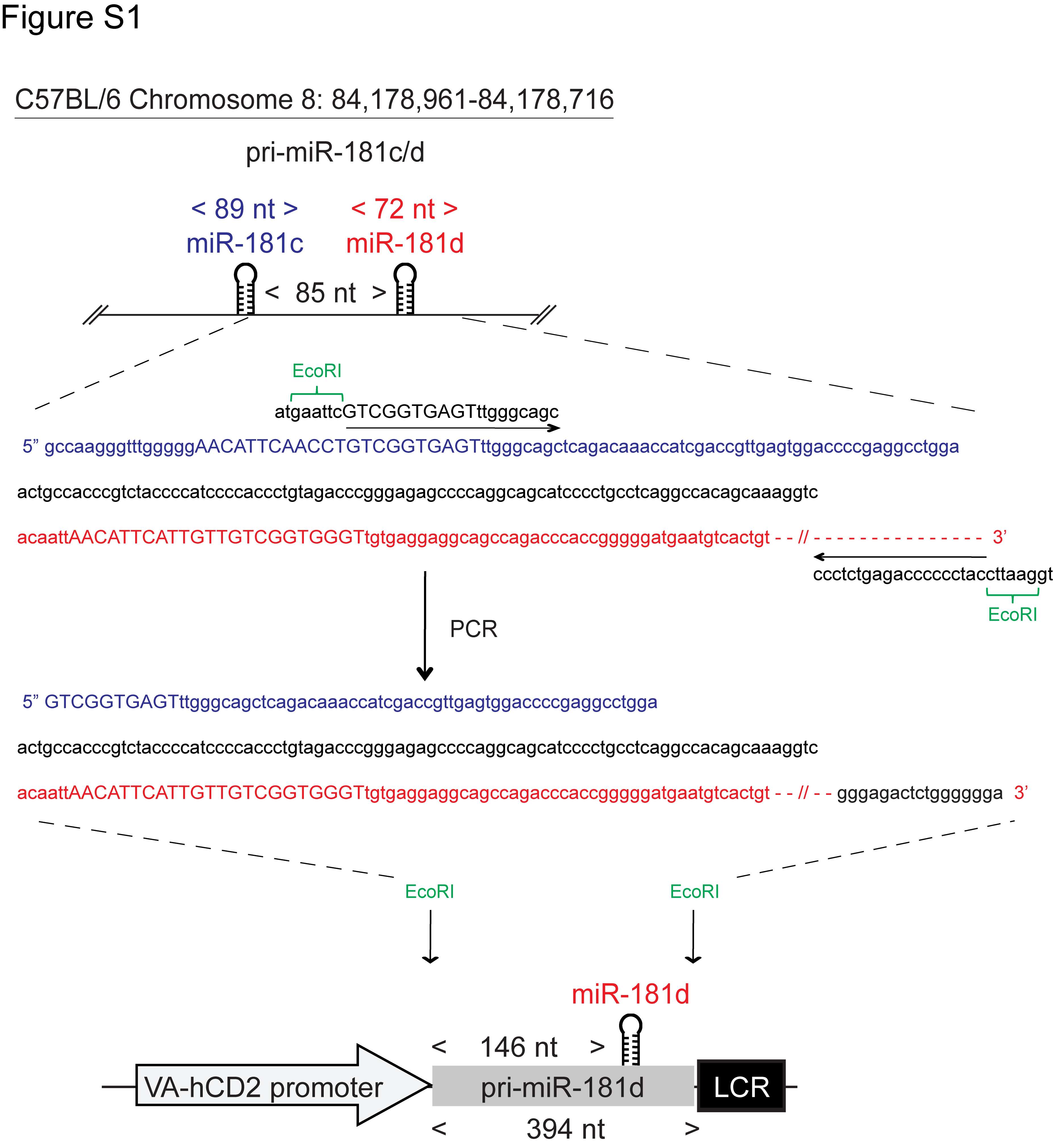

Supplement: Figure S1 — Generation of the VA-hCD2-pri-miR-181d transgenic cassette. Pri-miR-181c/d cluster is located on mouse chromosome 8. Lengths of miR-181c (blue) and miR-181d (red) sequences are 89 and 72 nucleotides, respectively. A 394 nt region containing whole pri-miR-181d and a 61nt portion of pri-miR-181c (lacking the seed sequence) was PCR amplified using the primers indicated with arrows and cloned into the VA-hCD2 transgenic cassette through EcoRI sites. Mature miR-181c (blue) and miR-181d (red) sequences are shown in uppercase, indicating that mature miR-181c sequence was excluded from the transgenic cassette. (TIF) [file pone.0085274.s001.tif]

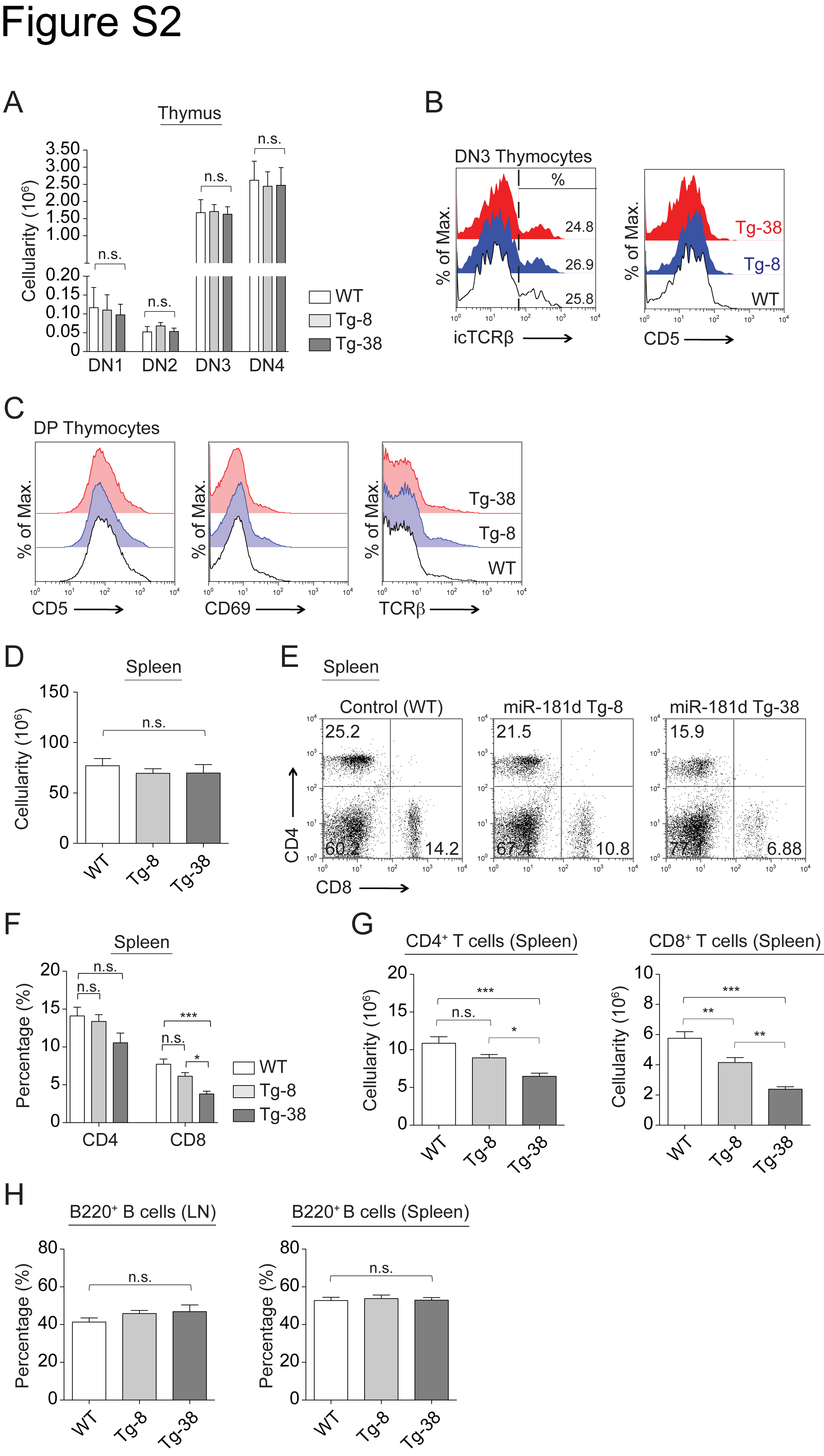

Supplement: Figure S2 — Characterization of lymphocytes in miR-181d transgenic mice. (A) CD25 and CD44 markers were used to define DN subsets by gating on CD4− CD8− B220− NK1.1− TCRγδ− CD11b− and CD11c− thymocytes. Absolute numbers of DN subsets are shown as the mean +/− SEM using at least 6 mice per group. (B) Histograms show intracellular TCRβ and surface CD5 expression in DN3 thymocytes from the WT (white), Tg-8 (blue), and Tg-38 (red) mice. Average percentages of intracellular TCRβ+ DN3 thymocytes are provided. (C) Histograms show CD5, CD69, and TCRβ expression on DP thymocytes. (D) Total cellularity in the spleen of the control and miR-181d Tg mice. (E) Representative FACS plots show CD4 by CD8 profiles in the spleen. (F–G) Average percentages (F) and absolute numbers (G) of CD4+ and CD8+ T cells in the spleen. (D–G) Data are from the WT (n = 16), Tg-8 (n = 16), and Tg-38 (n = 11) mice. (H) Average percentages of B220+ B cells in the lymph nodes (left) and spleen (right) using at least 10 mice per group. All bar graphs show the mean +/− SEM (n.s. = non-significant, *p<0.05, **p<0.01, ***p<0.001; One-way ANOVA followed by Tukey's post-hoc test). (TIF) [file pone.0085274.s002.tif]

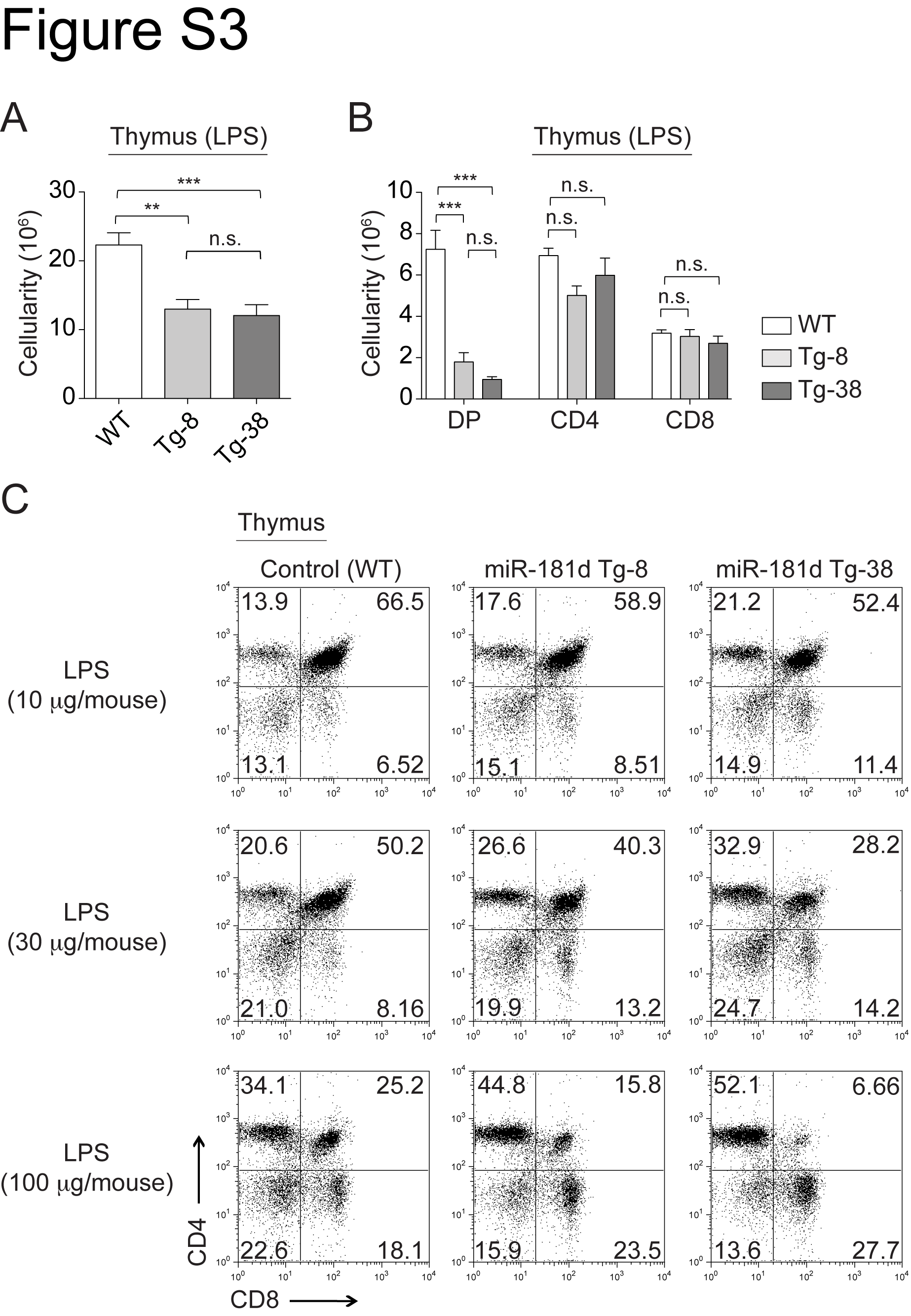

Supplement: Figure S3 — Stress-induced thymic atrophy in miR-181d transgenic mice. (A) Total thymic cellularity in the control and miR-181d Tg mice at 72 hours upon LPS injection (100 µg/mouse). (B) Absolute cellularity of thymocyte subsets (DP, CD4 SP, and CD8 SP) after 72 hours post-LPS injection. (A–B) Data are of the mean +/− SEM from at least 4 independent experiments using at least 3 mice per treatment (n.s. = non-significant, *p<0.05, **p<0.01, ***p<0.001; One-way ANOVA followed by Tukey's post-hoc test). (C) FACS plots show CD4 by CD8 profiles in the thymus of the control and miR-181d Tg mice at 72 hours upon LPS injection at varying concentrations (10, 30, and 100 µg/mouse). Percents are provided in each quadrant. (TIF) [file pone.0085274.s003.tif]

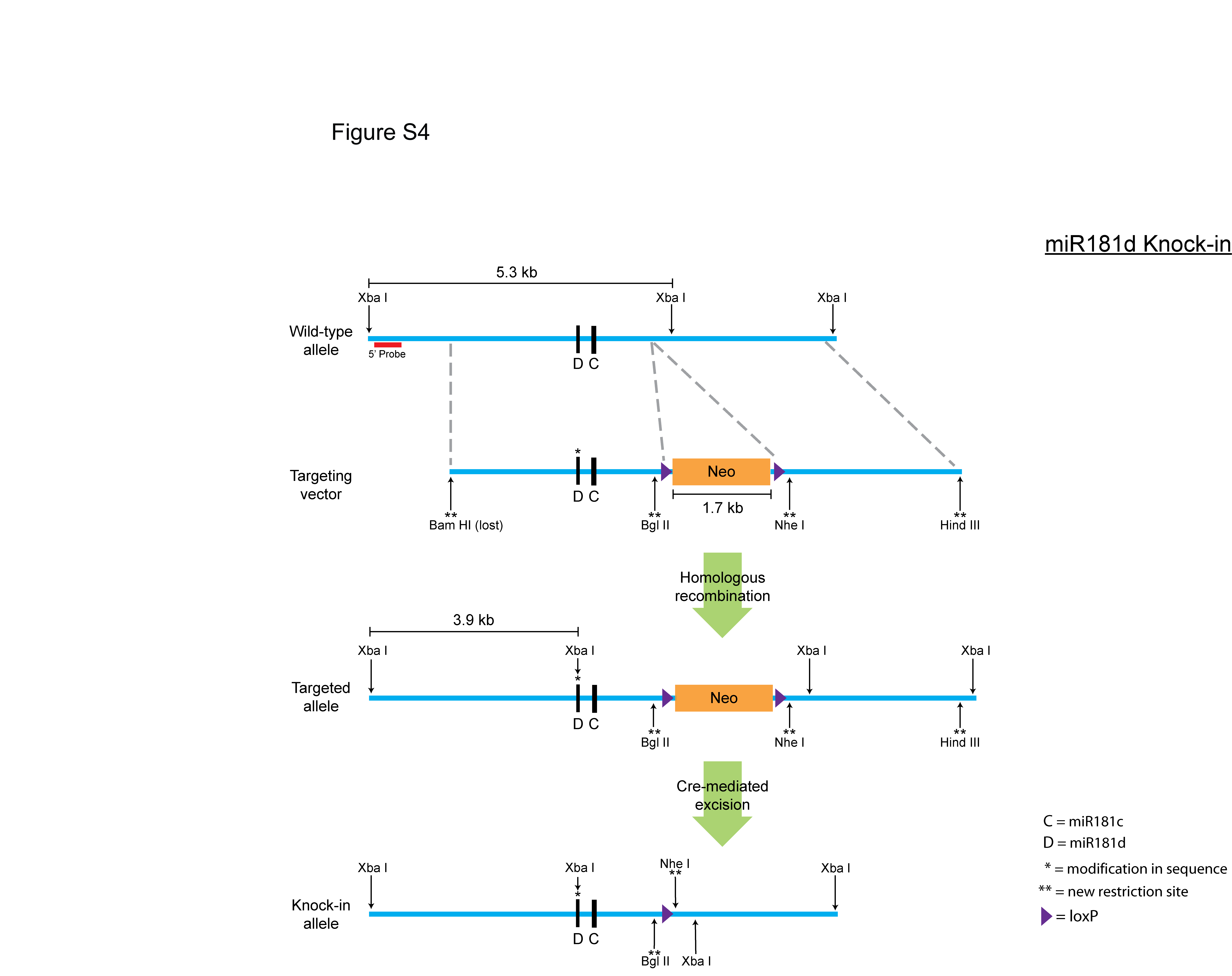

Supplement: Figure S4 — MiR-181d knock-in strategy. Schematic represents the generation strategy of miR-181d KI mice. (TIF) [file pone.0085274.s004.tif]

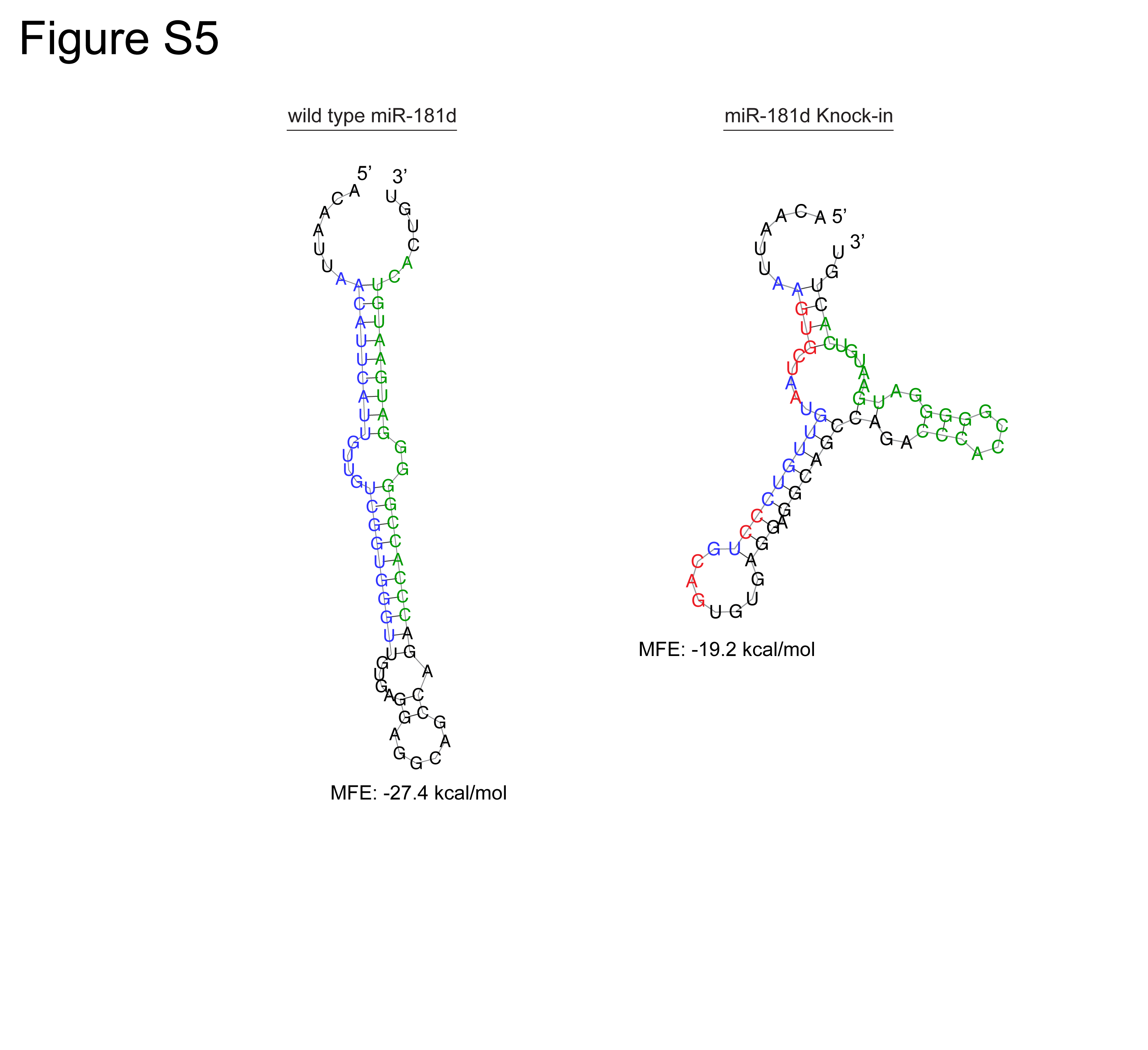

Supplement: Figure S5 — Predicted secondary structures of the wild-type miR-181d and miR-181d knock-in sequences. RNAfold Web Server (http://rna.tbi.univie.ac.at/cgi-bin/RNAfold.cgi) was used to obtain Minimum Free Energy (MFE) structures. Mature mir-181c and miR-181d sequences are highlighted in green and blue, respectively. Mutated bases in the miR-181d knockin sequence are highlighted in red. (TIF) [file pone.0085274.s005.tif]

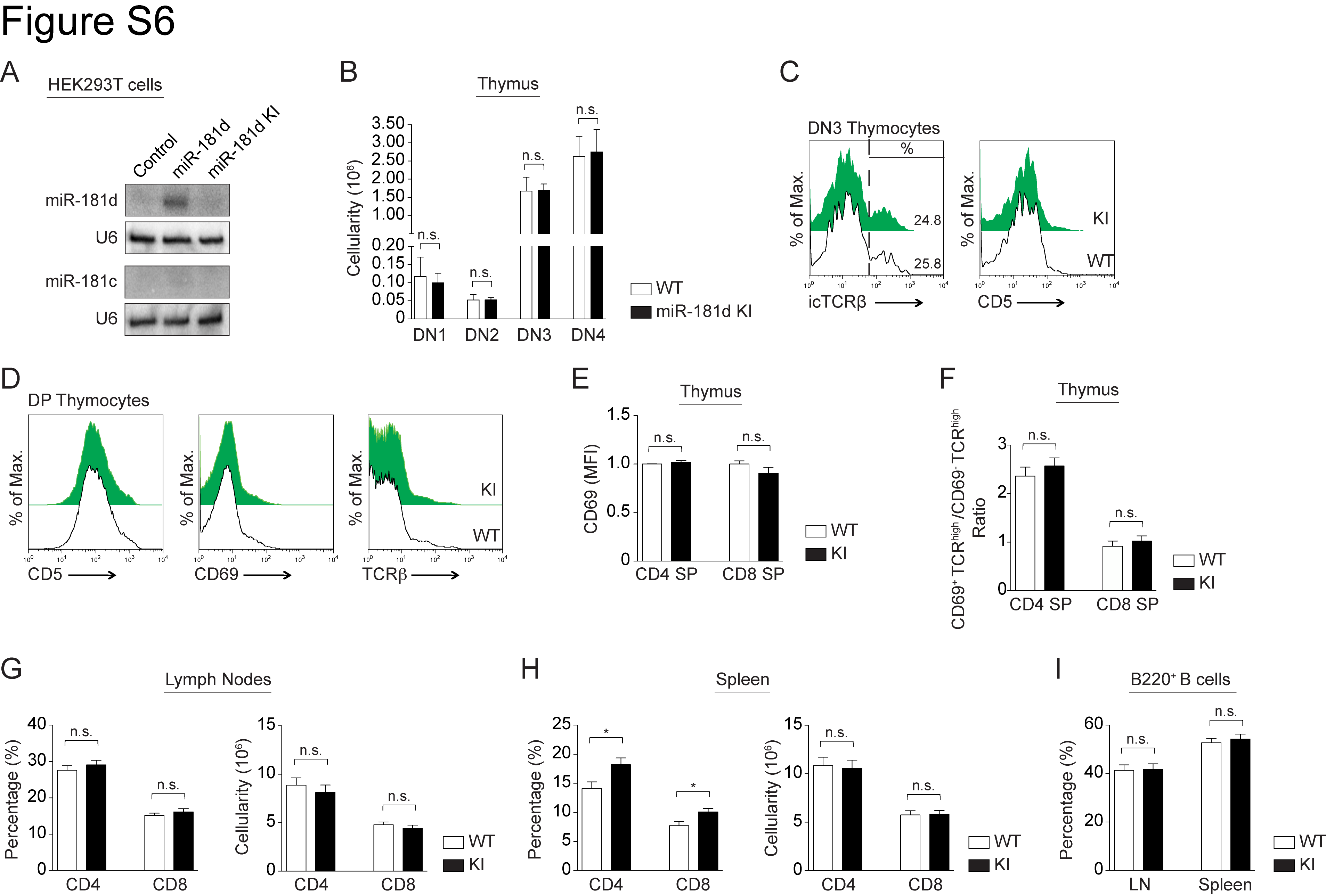

Supplement: Figure S6 — Characterization of miR-181d knock-in mice. (A) Northern blot shows miR-181d and miR-181c expression in HEK293T cells transfected with pCDNA3.1 control, pCDNA3.1/miR-181d, or pCDNA3.1/miR-181d KI plasmids. A U6 probe was used as endogenous control. Data are representative of 2 independent experiments. (B) Absolute numbers of DN thymocyte subsets in the thymus of the control and miR-181d KI mice. Data are of the mean +/− SEM using at least 6 mice per group (n.s. = non-significant; Two-tailed unpaired Student's t-test). (C) Histograms show intracellular TCRβ (icTCRβ) and surface CD5 expression in DN3 thymocytes from the WT (white) and miR-181d KI (green) mice. Average percentages of icTCRβ+ DN3 thymocytes were provided. (D) Histograms show CD5, CD69, and TCRβ expression gated on DP thymocytes from the WT (white) and miR-181d KI (green) mice. (E) Relative MFI (Mean Fluorescence Intensity) levels of CD69 on SP thymocytes. (F) Ratio of the CD69+TCRβhigh to CD69−TCRβhigh thymocyte numbers gated on CD4 SP and CD8 SP thymocytes. (E–F) Data show the mean +/− SEM values from at least 3 mice per group (n.s. = non-significant; Two-tailed unpaired Student's t-test). (G–H) Average percentages and absolute cell numbers of CD4+ T and CD8+ T cells in the lymph nodes (G) and spleen (H) of the WT (n = >16) and miR-181d KI (n = >13) mice. (I) Average percentages of B220+ B cells in the lymph nodes and spleen using at least 13 mice per group. All bar graphs show the mean +/− SEM (n.s. = non-significant; Two-tailed unpaired Student's t-test). (TIF) [file pone.0085274.s006.tif]
