# Supplementary material for: Transgenic Expression of MicroRNA-181d Augments the Stress-Sensitivity of CD4+CD8+ Thymocytes
Source: PLoS One. 2014 Jan 9;9(1):e85274. doi: 10.1371/journal.pone.0085274 (PMC3887031; doi:10.1371/journal.pone.0085274)
Supplement: Table S1 — List of down-regulated genes more than 1.5-fold in miR-181d Tg-38 thymus compared to the wild type control. (PDF) [file pone.0085274.s007.pdf]

Table S1. List of down-regulated genes more than 1.5-fold in miR-181d Tg-38 thymus compared to the wild type control.

Symbol	Avg WT	SD	Avg Tg-38	SD	Ratio (Tg-38/WT)	Accession	Probe_ID
Mid1	26	2	6	2	0.24	NM_183151.1	ILMN_3159435
Mpp5	6	1	2	1	0.31	NM_019579.1	ILMN_2594593
LOC333685	9	2	3	0	0.37	XM_289972.1	ILMN_2685383
5133401H10Rik	4	0	2	1	0.39	AK019892	ILMN_2551611
Npc1	5	1	2	1	0.39	AK044878	ILMN_1252918
LOC195284	6	1	2	2	0.4	XM_111584.2	ILMN_2528721
Rbl1	6	1	2	1	0.4	AK033402	ILMN_1240408
LOC333423	4	0	2	0	0.41	XM_286167.2	ILMN_2533496
5031439A09Rik	10	2	4	0	0.41	AK031444	ILMN_2554118
0610035N01Rik	4	1	2	0	0.42	AK030365	ILMN_1228112
Ppp2r5c	5	1	2	1	0.42	NM_012023	ILMN_2742461
Olfr244	6	0	3	1	0.42	NM_001005520.1	ILMN_3163040
Erdr1	236	9	103	39	0.43	NM_133362.1	ILMN_1246153
Zfp288	4	1	2	0	0.44	XM_147215.1	ILMN_1259424
D030006P03Rik	4	1	2	1	0.44	AK083427	ILMN_2577999
4832420L08Rik	39	3	17	3	0.44	AK029315	ILMN_1238535
Il17re	5	1	2	1	0.44	NM_001034031.1	ILMN_3162860
2900054C01Rik	4	0	2	1	0.45		ILMN_2438962
F830002E14Rik	143	35	65	10	0.45	AK089567	ILMN_1215076
1110036O03Rik	17	2	8	1	0.46	NM_176830.2	ILMN_1231457
LOC330240	3	1	2	1	0.46	XM_285905.2	ILMN_1216554
Mnt	7	1	3	0	0.47	NM_010813	ILMN_1236972
B430205M18Rik	4	1	2	1	0.47	AK046612	ILMN_1239902
Ifi203	4	0	2	1	0.47	NM_008328.1	ILMN_1241443
BC048403	5	1	2	0	0.47	NM_173022.2	ILMN_2596666
Mybl2	4	0	2	0	0.47	NM_008652.2	ILMN_2637886
Nf1	5	1	2	0	0.47	NM_010897.1	ILMN_1231104
Leprel2	8	2	4	0	0.47	NM_013534.1	ILMN_1239380
Zfp36l1	5	0	2	1	0.48	NM_007564.2	ILMN_2427108
Mad5	11	0	5	2	0.48	AK032570	ILMN_1222658
Entpd4	6	1	3	1	0.48	NM_026174	ILMN_1226174
D930049F02Rik	8	1	4	1	0.48	AK086749	ILMN_1233004
Plod2	14	3	7	1	0.49	NM_011961	ILMN_2770429
Rcbtb2	7	1	3	1	0.49	NM_134083.2	ILMN_2812087
Wbscr27	12	1	6	2	0.49	NM_024479.1	ILMN_1235834
Il4	6	1	3	1	0.49	NM_021283.1	ILMN_2931334
Smbp	4	0	2	1	0.49	NM_133352	ILMN_2603545
2210404G23Rik	3	1	2	1	0.5	NM_025958.1	ILMN_2619869
4921521J11Rik	4	0	2	1	0.5	NM_027590	ILMN_1225889
Abhd3	4	0	2	1	0.5	NM_134130.1	ILMN_2677728
Sprn	4	0	2	0	0.5	NM_183147	ILMN_2634644
C230060D12Rik	5	1	2	1	0.5	AK082535	ILMN_2580105
Il17re	36	2	20	3	0.54	NM_145826.2	ILMN_2634933
Il17re	34	6	18	3	0.54	NM_001034029.1	ILMN_3163006
LOC240539	23	5	13	1	0.54	XM_140607.1	ILMN_1230605
B230387C07Rik	57	6	31	1	0.55	AK046455	ILMN_1216085
A630040A01Rik	27	5	15	3	0.55	AK041811	ILMN_2561533

Cnn3	100	14	55	9	0.55	NM_028044.1	ILMN_2589785
Fgfbp3	20	3	11	1	0.56	NM_028263.1	ILMN_2841593
2310047L21Rik	57	3	32	1	0.56	AK009885	ILMN_1260397
Rab9	25	5	14	2	0.57	NM_019773.1	ILMN_2783918
Ptms	38	5	22	3	0.58	NM_026988.1	ILMN_2918838
Gadd45a	42	6	25	3	0.58	NM_007836.1	ILMN_2947568
Lig4	37	4	21	4	0.58	NM_176953.2	ILMN_2701654
Xkrx	71	5	42	4	0.59	NM_183319.2	ILMN_3161225
Dusp10	71	13	42	4	0.59	NM_022019.2	ILMN_2818964
Dhcr24	50	7	29	2	0.59	XM_131538.1	ILMN_2747031
Bach1	149	27	88	24	0.59	NM_007520.1	ILMN_2741677
Slc7a11	89	12	53	4	0.59	NM_011990.2	ILMN_2948143
Ppp1r3b	23	2	14	2	0.59	NM_177741.2	ILMN_2626098
Dclre1c	26	0	15	5	0.6	NM_146114.1	ILMN_1240123
Bcl2l1	96	14	57	3	0.6	NM_009743.2	ILMN_2698430
6330406I15Rik	23	2	14	1	0.6	NM_027519.1	ILMN_2642517
Sla	87	3	52	2	0.6	NM_009192	ILMN_1216337
Tube1	73	14	44	3	0.6	XM_125543.4	ILMN_1250535
E130007O11Rik	23	2	14	1	0.6	AK087403	ILMN_1213840
6430601A21Rik	42	8	25	3	0.61	NM_175466.2	ILMN_1217060
Plekhf1	30	3	18	2	0.61	NM_024413.1	ILMN_2993334
Dusp6	262	46	162	10	0.62	NM_026268.1	ILMN_2925711
Fbxl12	95	8	58	7	0.62	NM_013911.1	ILMN_1224697
BC011210	20	3	12	2	0.62	NM_173863.1	ILMN_1248891
Chd2	86	17	53	6	0.62	XM_145698.4	ILMN_1213322
6330415F13Rik	38	1	23	2	0.62	NM_027533	ILMN_1234698
Rasa2	115	16	72	9	0.62	NM_053268	ILMN_2715328
Cd79b	43	2	27	2	0.63	NM_008339.1	ILMN_2661523
B930006L02Rik	20	2	13	1	0.63	NM_178764.3	ILMN_2623112
Tnfrsf1a	21	3	13	3	0.63	NM_011609.2	ILMN_2469333
D330026F23Rik	46	6	29	2	0.63	AK084659	ILMN_2579391
Egln2	21	2	13	3	0.63	NM_053208.2	ILMN_2781805
Zfpn1a2	26	2	16	2	0.63	NM_011770.2	ILMN_1217123
Tgfb1i4	31	5	20	4	0.63	NM_009366.1	ILMN_2723108
Vil2	453	72	290	13	0.64	NM_009510	ILMN_1232495
A530089A20Rik	259	38	165	23	0.64	AK041191	ILMN_2568488
Dusp4	51	1	33	5	0.64	NM_176933	ILMN_2450767
LOC383153	27	4	17	3	0.64	XM_356899.1	ILMN_2537361
Chd9	60	4	38	3	0.64	XM_284439	ILMN_2697954
Cyb5	219	27	141	18	0.64	NM_025797.1	ILMN_2966632
Sox4	460	41	298	17	0.65	NM_009238	ILMN_2727116
LOC383077	108	19	70	10	0.65	XM_356845.1	ILMN_2536687
Lef1	73	2	48	7	0.65	NM_010703	ILMN_2648475
Cutl1	26	3	17	2	0.65	NM_009986.2	ILMN_1229977
LOC212399	60	6	39	6	0.65	XM_137003.2	ILMN_1244312
2700049H19Rik	37	6	24	3	0.65		ILMN_1229329
Mll5	130	17	85	6	0.65	XM_485570	ILMN_1217776
Ppa2	34	5	23	4	0.65	NM_146141.1	ILMN_1234589
Ltb	194	18	127	8	0.65	NM_008518.1	ILMN_1258283
Dgkg	140	7	92	8	0.66	NM_138650	ILMN_1237518

Chrna9	116	8	76	7	0.66	XM_132045.2	ILMN_2717580
Sypl	198	4	130	14	0.66	NM_013635	ILMN_2758720
Hes6	32	1	21	2	0.66	NM_019479.2	ILMN_1214126
Hist1h4k	35	4	23	1	0.66	NM_178211.1	ILMN_1243654
4930422J18Rik	67	6	44	6	0.66	NM_029035.2	ILMN_1224336
Slc9a9	46	3	30	6	0.66	NM_177909.2	ILMN_2637113
Runx1	64	8	42	4	0.66	NM_009821	ILMN_2589107
Nphs2	61	5	40	4	0.66	NM_130456.2	ILMN_2717146
Chd1	106	16	70	8	0.66	AK021188	ILMN_1254296
Camkk1	72	1	48	3	0.66	NM_018883.1	ILMN_1242310
Chd4	34	6	22	2	0.67	NM_145979.1	ILMN_2654822
F13a1	212	19	141	11	0.67	NM_028784.2	ILMN_2914938
Hist1h4c	23	2	15	0	0.67	NM_178208.1	ILMN_2588244
Dscr3	23	3	15	1	0.67	NM_007834.1	ILMN_2988631
