# Supplementary material for: Transgenic Expression of MicroRNA-181d Augments the Stress-Sensitivity of CD4+CD8+ Thymocytes
Source: PLoS One. 2014 Jan 9;9(1):e85274. doi: 10.1371/journal.pone.0085274 (PMC3887031; doi:10.1371/journal.pone.0085274)
Supplement: Table S2 — List of up-regulated genes more than 1.5-fold in miR-181d Tg-38 thymus compared to the wild type control. (PDF) [file pone.0085274.s008.pdf]

Table S2. List of up-regulated genes more than 1.5-fold in miR-181d Tg-38 thymus compared to the wild type control.

Symbol	Avg WT	SD	Avg Tg-38	SD	Ratio (Tg-38/WT)	Accession	Probe_ID
5031439A09Rik	25	1	164	12	6.432227176	NM_026582	ILMN_2704186
Gpr177	51	9	280	37	5.509818961	NM_026582.3	ILMN_2996561
Tlr1	3	1	12	3	4.817682379	NM_030682.1	ILMN_1236908
Klrd1	25	4	119	24	4.798532756	NM_010654.1	ILMN_1255860
V1rd6	2	0	8	2	4.796771918	NM_030738.1	ILMN_2971043
Cxcl9	9	2	35	6	3.948071884	NM_008599	ILMN_1215862
Rab4a	4	1	15	5	3.942609957	NM_009003.1	ILMN_1212902
F2rl1	14	5	56	7	3.880890098	NM_007974	ILMN_2769567
Gpr68	13	1	44	13	3.457300024	NM_175493.2	ILMN_2732996
0610005K03Rik	2	2	8	1	3.394022323	XM_128291.3	ILMN_1248788
LOC385615	9	2	31	5	3.393459347	XM_358790.1	ILMN_1218717
Serpina3h	2	1	6	1	3.304124077	NM_001034870.2	ILMN_2889832
Mmp13	3	1	10	1	3.27555415	NM_008607.1	ILMN_2737685
Cxcr6	2	1	7	2	3.174954095	NM_030712.1	ILMN_1221846
Gp2	3	2	10	1	3.171454828	NM_025989.1	ILMN_2904435
Rgs11	2	1	8	2	3.121714332	XM_128488.3	ILMN_2667199
Ttn	2	1	5	1	3.119298495	NM_028004.2	ILMN_3128792
Fezf2	2	1	7	2	3.101517105	NM_080433.1	ILMN_2932692
Gpr114	72	9	217	16	2.989124644	NM_001033468.1	ILMN_2856926
Crhbp	2	1	6	1	2.942907545	NM_198408.1	ILMN_1231710
Mucdhl	2	1	6	2	2.924644814	NM_028069.1	ILMN_2749556
Expi	8	1	23	5	2.880741008	NM_007969.2	ILMN_1239717
0610041G09Rik	9	4	26	4	2.877954098	NM_183274	ILMN_2693895
1700040A22Rik	4	2	10	2	2.856126544		ILMN_1246757
Folr4	2	0	5	0	2.842131615	NM_176807.2	ILMN_2659388
Slc6a4	2	1	5	2	2.839027231	AK078634	ILMN_2585676
Igh-1a	20	5	55	13	2.746464752	XM_354704.1	ILMN_2633179
Rptn	5	3	14	0	2.74475159	NM_009100.1	ILMN_2667665
4833405L16Rik	2	1	5	1	2.741663248	NM_177197.2	ILMN_1216803
E430019B13Rik	2	1	5	2	2.735684918	NM_172842.2	ILMN_2668455
Havcr2	2	1	6	1	2.735596975	NM_134250.1	ILMN_2721929
D430044G18Rik	5	2	15	3	2.73337498	NM_172628	ILMN_1240728
Ttc8	2	1	5	1	2.712319941	NM_198311.1	ILMN_3141738
Clcf1	2	0	4	1	2.709265385	NM_019952.1	ILMN_2926729
C030009J22Rik	2	1	6	1	2.64626957		ILMN_2463583
Slc1a2	2	1	4	1	2.63457163	NM_001077514.1	ILMN_3047516
Rgs17	2	1	4	1	2.633365047	NM_019958	ILMN_1219289
Inmt	5	3	14	3	2.613131742	NM_009349.1	ILMN_2803249
Klra18	2	1	5	1	2.611725353	NM_053153.1	ILMN_1252295
Flg	3	1	8	1	2.60791476		ILMN_2455468
Fabp9	3	2	7	0	2.552552811	NM_011598.2	ILMN_1221705
4933426K21Rik	2	0	5	0	2.539350539	AK016936	ILMN_1247736
Krt1-23	2	1	6	1	2.536934275	NM_033373.1	ILMN_2671165
Klra7	3	1	8	1	2.51071401	NM_014194.1	ILMN_1227867
Haao	5	0	13	1	2.476723804	XM_354652	ILMN_2607951
Rgs3	3	1	7	2	2.461584018	NM_019492	ILMN_1241709
E430002D04Rik	37	3	90	12	2.459417806	NM_172909.1	ILMN_2629663

BC023892	7	1	17	0	2.439409125	XM_135029	ILMN_2502471
Ccl4	25	7	62	4	2.436544006	NM_013652.1	ILMN_1223257
Rab25	2	0	6	1	2.422053687	NM_016899	ILMN_2776087
Ear6	3	0	6	1	2.419298966	NM_053111.2	ILMN_2871749
Ccl21a	25	10	61	20	2.417315718	NM_011335.1	ILMN_2602139
Dmkn	2	1	6	1	2.392062243	NM_172899.2	ILMN_1229763
Mab21l2	2	0	5	1	2.390821214	NM_011839.2	ILMN_2595346
Ifit3	54	10	128	14	2.379986566	NM_010501.1	ILMN_2944666
Syt1	5	3	13	1	2.378897599	NM_009306	ILMN_1224963
Actn2	51	3	120	11	2.377587506	NM_033268.3	ILMN_2764727
Doc2g	2	1	4	1	2.346727778	NM_021791.2	ILMN_2704429
Grasp	36	2	85	12	2.335730877	NM_019518.2	ILMN_2596396
Pcsk4	2	1	5	1	2.324277385	NM_008793.1	ILMN_2629776
Cyp2a5	9	4	21	4	2.314674473	NM_007812.1	ILMN_3159131
Eppk1	5	3	12	2	2.309183804	NM_144848.2	ILMN_1243900
1700048O20Rik	5	2	12	2	2.308789661		ILMN_2479690
B830021E24Rik	46	5	106	14	2.306393526	NM_198034	ILMN_1248211
Nkg7	69	5	158	40	2.305416588	NM_024253.3	ILMN_1217855
Padi1	4	2	9	1	2.300495751	NM_011059.1	ILMN_2637742
Grifin	8	1	18	3	2.289219398	XM_132470.1	ILMN_2676543
Klk7	2	1	4	0	2.272605376	NM_011872.2	ILMN_1251558
Ly6c	42	5	96	15	2.271728092	NM_010741	ILMN_1254927
Cyp2s1	5	2	10	2	2.251382995	NM_028775.2	ILMN_2758264
Mx1	2	1	4	1	2.251354578	NM_010846	ILMN_2707870
Spink3	15	10	34	3	2.249422959	NM_009258.2	ILMN_2708477
A930033C01Rik	2	1	4	1	2.245221134	XM_132396.4	ILMN_1244474
Adam19	24	2	53	12	2.236498585	NM_009616	ILMN_2772155
Knsl1	2	1	5	1	2.232809636	AK050778	ILMN_1251822
EG433016	2	1	5	1	2.225226993	NM_001082547.1	ILMN_2995688
Cd86	41	6	91	10	2.216275774	NM_019388.2	ILMN_2743244
Dnaja4	3	1	6	2	2.215278008	NM_021422.2	ILMN_2689986
Gjb2	4	1	8	1	2.210591251	NM_008125.2	ILMN_2999627
Art2b	2	1	4	1	2.200586669	NM_019915.1	ILMN_1215074
2210023G05Rik	3	0	6	0	2.196393892	NM_197999.1	ILMN_2895284
Mylk	2	0	4	1	2.191760791	NM_139300	ILMN_1219471
4930545K18Rik	2	1	4	1	2.189081097		ILMN_2455063
Xcl1	21	5	45	8	2.181765753	NM_008510.1	ILMN_2948552
Ccl21b	55	18	120	26	2.179000259	NM_011124.3	ILMN_1213286
Bok	4	1	9	1	2.178332381	NM_016778	ILMN_2772077
2410014A08Rik	4	0	8	1	2.177641125	NM_175403.2	ILMN_2997796
Golph2	13	2	28	4	2.173210289	NM_027307	ILMN_2623536
BC011468	7	3	16	3	2.170248639	NM_144847.1	ILMN_2594031
7420404O03Rik	73	2	157	6	2.158486708	AK078662	ILMN_1239863
Golph2	10	1	21	2	2.15411843	NM_001035122.2	ILMN_3148550
1200009I06Rik	3	1	6	1	2.153327417	NM_028807.1	ILMN_2740207
Temt	40	18	86	17	2.152063347	NM_009349	ILMN_1231445
Ccl19	2	1	5	1	2.15035937	NM_011888.2	ILMN_2836386
E130013N09Rik	19	6	42	7	2.15002803	XM_488964	ILMN_1238627
Gnmt	2	0	3	0	2.142335914	NM_010321.1	ILMN_2837816
Cd7	4	1	8	2	2.128808824	NM_009854.1	ILMN_1252079

D030029J20Rik	2	1	4	1	2.126232509		ILMN_1257291
Sct	6	1	12	3	2.12579819	NM_011328.1	ILMN_2592834
5830467P10Rik	4	1	9	1	2.125778449	NM_198029.1	ILMN_1234099
H1f0	12	2	26	4	2.12255221	NM_008197.2	ILMN_1259559
Tle6	41	6	86	10	2.115747559	NM_053254.2	ILMN_2900617
Ctsw	154	15	324	51	2.105533866	NM_009985.2	ILMN_2721399
E330039G21Rik	4	1	8	1	2.104968415	XM_203357.3	ILMN_2667190
Rhpn1	2	1	4	1	2.099025439	NM_008164	ILMN_2746985
B430007K19Rik	2	0	3	0	2.096879297		ILMN_2446700
2410012C07Rik	18	6	37	3	2.094565085	XM_133920.3	ILMN_2757844
Il6ra	23	4	49	9	2.093147322	AK020663	ILMN_1255743
Prkg2	2	0	4	1	2.092897138	NM_008926.2	ILMN_3160326
Herc3	2	1	4	1	2.089156429	NM_028705.2	ILMN_2635218
Pla1a	2	1	5	1	2.08636703	NM_134102.2	ILMN_2974343
Tbx21	2	1	5	1	2.078552126	NM_019507.1	ILMN_2762983
4930431B09Rik	7	0	15	1	2.077168288	XM_207778.3	ILMN_2684205
9830143E02Rik	2	1	5	0	2.076703068	AK036626	ILMN_1219232
1700024K14Rik	2	0	4	1	2.076478563	AK075758	ILMN_1241150
G430091H17Rik	7	3	14	1	2.076155672	AK090072	ILMN_1229197
Chi3l1	7	1	15	4	2.074484162	NM_007695.1	ILMN_2609813
Krt1-14	23	9	48	11	2.07398478	NM_016958.1	ILMN_2722616
Trim2	2	1	4	1	2.068727096	NM_030706.1	ILMN_2511355
Fjx1	2	1	5	1	2.06791993	NM_010218	ILMN_2689998
D11Ertd686e	2	1	3	1	2.062505338	XM_110968	ILMN_2630138
Idi1	2	0	4	1	2.062424961	NM_145360	ILMN_2590923
1600023A02Rik	13	5	27	4	2.058332178	NM_026323.1	ILMN_1236758
Abhd5	4	1	8	1	2.055273948	NM_026179.1	ILMN_2739295
Ly116	12	8	25	2	2.05391389	AK080934	ILMN_1241598
Oas2	27	5	56	8	2.05336191	NM_145227.1	ILMN_2670150
D630040I23Rik	17	1	36	4	2.051357773		ILMN_2495167
Chk	3	0	5	1	2.042214368	AK053818	ILMN_1255524
Gfi1b	3	2	5	1	2.040366436	NM_008114.2	ILMN_2895557
1700019H03Rik	11	2	22	5	2.034692714	NM_183161.3	ILMN_2999818
C330015L04Rik	3	1	6	1	2.030210844	AK049236	ILMN_1214565
Ank	8	2	16	1	2.027697108	NM_020332.2	ILMN_1232621
9530008L14Rik	2	0	3	0	2.023042463	NM_175417.2	ILMN_2962041
Oas1b	5	2	11	2	2.021816963	NM_011853.1	ILMN_2613140
Dip3b	21	6	43	4	2.021033715	NM_145220.1	ILMN_1219978
AW536289	38	5	77	11	2.015309278		ILMN_1251178
Asns	4	0	9	2	2.014004554	NM_012055.1	ILMN_2643513
Cyp27a1	3	1	7	1	2.008356198	NM_024264.3	ILMN_2620326
2310015J09Rik	2	0	3	1	2.005994378	NM_027983.1	ILMN_1235868
Pglyrp1	23	5	45	9	1.984884257	NM_009402.1	ILMN_2592486
Card10	15	1	29	2	1.971761961	NM_130859.2	ILMN_2606660
Il7r	95	10	186	24	1.964576355	NM_008372.3	ILMN_2680827
Gpr18	74	15	144	25	1.942620903	NM_182806.1	ILMN_1213809
Itgb7	100	2	192	34	1.918025402	NM_013566.1	ILMN_1227434
Cbr2	34	9	66	11	1.91472794	NM_007621.1	ILMN_2756665
Cd226	12	0	23	2	1.894863825	NM_178687	ILMN_1239753
LOC270152	23	2	44	4	1.874122179	XM_194453.3	ILMN_1231204

Ncf4	26	3	48	5	1.870122028	NM_008677.1	ILMN_2743013
Hdc	32	10	60	10	1.855994377	NM_008230.4	ILMN_2965903
Cd160	24	3	45	5	1.847454747	NM_018767.2	ILMN_2707181
2700038N03Rik	27	5	50	4	1.842023466	NM_027356.1	ILMN_2605703
Tcea2	24	2	43	5	1.832461972	NM_009326	ILMN_2777609
A730063M14Rik	13	3	23	1	1.821847678		ILMN_2509830
Zfp312	28	7	52	4	1.819875572	NM_080433	ILMN_2512430
BC038822	13	1	23	3	1.816744079	NM_172293.2	ILMN_1237625
Amica1	20	4	36	2	1.815258937	NM_001005421.2	ILMN_3160218
St5	50	15	90	5	1.809002784	NM_029811.1	ILMN_3154820
Slfn1	85	5	153	19	1.803248193	NM_011407.1	ILMN_2663930
1110007C02Rik	25	2	45	7	1.800530987	NM_027923.1	ILMN_1225374
A930034L06Rik	19	5	33	3	1.795768881	NM_175692.3	ILMN_2952098
B930041F14Rik	29	3	52	7	1.779650259	NM_178699	ILMN_1250201
Gpnmb	12	2	22	4	1.779291379	NM_053110.2	ILMN_2648669
Ppic	39	9	69	8	1.765547608	NM_008908.1	ILMN_2810882
Itgae	95	8	168	28	1.764554328	NM_008399.1	ILMN_1217629
Rab15	13	4	23	2	1.763331665	NM_134050.2	ILMN_1217009
Irf7	14	3	25	4	1.76277305	NM_016850.1	ILMN_1227573
AI451557	53	5	93	17	1.761203083	NM_001033207.1	ILMN_3161790
Samhd1	83	17	145	22	1.752833319	NM_018851.2	ILMN_1221736
Aire	19	7	33	2	1.748489735	NM_009646.1	ILMN_2707921
Siat10	83	11	145	10	1.744608268	NM_018784	ILMN_2695199
Tmie	46	5	80	5	1.735747291	NM_146260.1	ILMN_1218037
AA960558	114	7	198	30	1.733730818	NM_133942.1	ILMN_2708877
Ddr1	14	4	25	4	1.713013405	NM_007584.1	ILMN_2713898
Gcat	21	4	36	1	1.70566937	NM_013847	ILMN_2777696
Ly6a	168	14	286	38	1.704984734	NM_010738.2	ILMN_1255416
H19	49	5	84	14	1.701167992	NR_001592.1	ILMN_1256343
Stag3	18	2	31	7	1.696591775	NM_016964.1	ILMN_2993843
Scarf2	56	15	95	7	1.690798658	NM_153790.1	ILMN_2676127
Nfix	22	1	38	1	1.680093241	NM_010906.1	ILMN_2742599
BC013481	19	2	31	4	1.673543199	NM_178446.2	ILMN_2720836
Il4i1	67	13	112	18	1.666117215	NM_010215.1	ILMN_2733778
Bteb1	14	4	23	1	1.663875969	NM_010638.2	ILMN_1254031
Prss19	142	15	236	31	1.660596058	NM_008940.1	ILMN_2675232
Fcgrt	34	4	56	10	1.660006457	NM_010189	ILMN_1221819
mt-Nd5	33	9	55	9	1.658883068		ILMN_2504544
5830431A10Rik	27	5	45	5	1.65344084	XM_488664	ILMN_2729252
Lrrc59	15	1	25	3	1.652474177	NM_133807.1	ILMN_1241854
Spint1	48	13	79	2	1.649648343	NM_016907	ILMN_1237186
Ifi30	13	4	22	3	1.648352877	NM_023065.2	ILMN_2767918
Eng	13	1	22	2	1.647027783	NM_007932	ILMN_1229161
9930108O06Rik	16	6	26	1	1.642741583	AK037075	ILMN_2566321
2210411K11Rik	30	2	49	4	1.638815135	XM_355846	ILMN_2736690
H2-M2	106	29	173	17	1.636633664	NM_008204.2	ILMN_2964185
Serpina3g	45	7	74	11	1.63621241	XM_354694.1	ILMN_2725927
Gprc5b	12	2	20	1	1.634941421	NM_022420.1	ILMN_2611767
Tnfaip8l2	21	3	34	4	1.634274919	NM_027206.1	ILMN_1249688
BC035954	13	3	21	4	1.634047139	NM_177569.2	ILMN_2759853

Tcea2	20	2	33	2	1.633384527	NM_009326.1	ILMN_2884728
A630023P12Rik	42	4	69	6	1.631876509	NM_173766.2	ILMN_2644193
AI132321	28	2	45	1	1.622007697	NM_178911.2	ILMN_1234223
Slco2b1	32	8	51	6	1.617660299	NM_175316.2	ILMN_2619707
3010031K01Rik	22	6	36	3	1.616438332		ILMN_2522460
Gpr155	16	2	26	5	1.613616935	XM_130346	ILMN_1249003
A930005H10Rik	65	1	104	4	1.607752499		ILMN_1250135
Nt5e	19	3	30	1	1.60301515	NM_011851.2	ILMN_2813830
1700021K02Rik	118	20	189	25	1.597385856	NM_023064	ILMN_2651010
Ctsh	41	2	65	8	1.5960191	NM_007801.1	ILMN_2872058
Rnf135	28	2	45	6	1.591314094	NM_028019.1	ILMN_2820379
Hvcn1	35	5	56	5	1.59009142	NM_001042489.1	ILMN_3124528
2810001G20Rik	61	8	95	6	1.577072845		ILMN_2431398
Sv2a	17	1	28	1	1.577015527	NM_022030.2	ILMN_2702887
Sema4a	149	7	235	47	1.574300007	NM_013658.2	ILMN_1215120
Alg8	45	2	71	1	1.568661563	NM_199035	ILMN_1237963
Ng23	41	3	65	14	1.565869848	XM_358322.1	ILMN_1239632
2610318I01Rik	32	5	50	5	1.56136272	NM_153118.1	ILMN_2746132
Ifitm1	58	4	90	18	1.558511447	NM_026820.2	ILMN_1217180
0610039P13Rik	65	4	101	10	1.556751968	NM_028752	ILMN_1243451
Il2ra	17	3	26	2	1.555544239	NM_008367	ILMN_1233474
Lyzs	145	23	225	8	1.55468843	NM_017372	ILMN_1252076
Enc1	21	2	32	4	1.54864366	NM_007930.3	ILMN_1237886
Anxa3	31	3	49	9	1.548609786	NM_013470.1	ILMN_3135781
D14Ertd668e	57	6	88	6	1.546927245	NM_199015.1	ILMN_2827780
Slc16a6	23	6	36	4	1.543959439	NM_134038	ILMN_1258950
Sas	56	4	87	6	1.543571821	NM_025982	ILMN_2757599
Faah	37	1	56	7	1.541168662	NM_010173.2	ILMN_2766930
Cdkn1c	68	7	105	15	1.540457368	NM_009876.2	ILMN_2708203
Lta	14	3	21	1	1.539832493	NM_010735.1	ILMN_2780247
Card4	33	5	50	6	1.535871379	NM_172729.1	ILMN_1247710
4931431C02Rik	23	3	36	4	1.535815193	XM_486200	ILMN_2760963
Mettl1	21	1	32	2	1.535538579	NM_010792.1	ILMN_3001946
Ccr8	16	3	24	4	1.535268098	NM_007720.1	ILMN_2724465
Bcat1	20	5	30	2	1.531477565	NM_007532.2	ILMN_3131478
Hap1	14	2	21	1	1.524232408	NM_177981.1	ILMN_1244829
Agrn	80	20	122	16	1.521618126	NM_021604	ILMN_2674367
Glipr2	82	10	124	11	1.508969642	NM_027450	ILMN_2773900
Sorl1	25	5	37	5	1.504070601	NM_011436	ILMN_1249578
Itgb4	29	5	44	0	1.500752978	NM_133663.2	ILMN_3144575
