# Supplementary material for: Transgenic Expression of MicroRNA-181d Augments the Stress-Sensitivity of CD4+CD8+ Thymocytes
Source: PLoS One. 2014 Jan 9;9(1):e85274. doi: 10.1371/journal.pone.0085274 (PMC3887031; doi:10.1371/journal.pone.0085274)
Supplement: Table S3 — Top 10 transcription factors with predicted target motifs among differentially regulated genes in the wild type control versus miR-181d Tg-38 thymocytes based on the significance level. (PDF) [file pone.0085274.s009.pdf]

Table S3. Top 10 transcription factors with predicted target motifs among differentially regulated genes in the wild type control vs miR-181d Tg-38 thymocytes based on the significance level. This enrichment analysis was performed using at least 10 genes for each category with hyper-geometric statistical test and Benjamini & Hochberg multiple test adjustment.

**Down-regulated genes in miR-181d Tg thymocytes**			
**Transcription factors with target motifs**	**Number of genes**	**Enrichment ratio**	**Adjusted p-value**
SP1_GGGCGGR	159	4.79	1.02×10^{-59}
MAZ_GGGAGGRR	135	5.22	1.54×10^{-54}
LEF1_CTTTGT	123	5.52	4.73×10^{-52}
E12_CAGGTG	133	4.72	5.91×10^{-49}
FOXO4_TTGTTT	110	4.76	2.48×10^{-40}
AP4_CAGCTG	95	5.57	3.72×10^{-40}
FREAC2_RTAAACA	67	6.38	1.75×10^{-31}
NFAT_TGGAAA	92	4.36	1.25×10^{-30}
MYC_CACGTG	64	5.49	1.88×10^{-26}
MEIS1_TGACAGNY	57	6.14	6.06×10^{-26}
**Up-regulated genes in miR-181d Tg thymocytes**			
**Transcription factors with target motifs**	**Number of genes**	**Enrichment ratio**	**Adjusted p-value**
SP1_GGGCGGR	126	3.07	1.29×10^{-26}
E12_CAGGTG	106	3.04	4.62×10^{-22}
MYC_CACGTG	59	4.09	1.06×10^{-17}
MAZ_GGGAGGRR	89	2.78	3.85×10^{-16}
AP4_CAGCTG	69	3.27	6.78×10^{-16}
NFAT_TGGAAA	78	2.99	6.90×10^{-16}
ETS2_RYTTCCTG	56	3.72	2.58×10^{-15}
LEF1_CTTTGA	76	2.76	9.74×10^{-14}
FOXO4_TTGTTT	77	2.70	1.79×10^{-13}
AP1_TGANTCA	51	3.22	1.04×10^{-11}
